# Bridging practice and precision: a quantitative HER2 protein assay ready for clinical use in guiding trastuzumab deruxtecan therapy

**DOI:** 10.3389/fonc.2026.1747961

**Published:** 2026-04-27

**Authors:** Junmei Hao, Xiaochun Fei, Fangfang Zou, Qian Hu, Linlin Du, Liya Yu, Xuanye Bai, Shasha Bi, Qinghua Cao, Weishan Chen, Qun Dai, Tingting Guo, Hai Huang, Wenwei Jiang, Baohua Li, Lixia Li, Jingjing Liu, Tong Liu, Xiaoqin Liu, Jiahong Lyu, Yue Pan, Xiaoqing Shao, Fangrong Tang, Tingting Wang, Cuiping Zhang, Yongcun Zhu, Job Tien Chiang Liu, Nicole Salazar, Shukun Zhang, Jiandi Zhang, Xiaosong Chen, Chaofu Wang

**Affiliations:** 1 Department of Pathology, Yantai Affiliated Hospital of Binzhou Medical University, Yantai, China; 2 Department of Pathology, Ruijin Hospital, Shanghai Jiaotong University School of Medicine, Shanghai, China; 3 Department of Pathology, Weihai Municipal Hospital, Cheeloo College of Medicine, Shandong University, Weihai, China; 4 Department of Pathology, Henan Cancer Hospital, Zhengzhou, China; 5 Department of Pathology, Quanzhou First Hospital, Quanzhou, China; 6 Department of Pathology, The Third People’s Hospital of Hefei, Hefei Third Clinical College of Anhui Medical University, Hefei, China; 7 Department of Pathology, The Fifth Affiliated Hospital of Xinjiang Medical University, Wulumuqi, China; 8 Department of Pathology, Autonomous Prefecture People’s Hospital, Xiangxi, China; 9 Yantai Zestern Biotechnique Co., LTD, A Division of Quanticision Diagnostics, Inc (US), Yantai, China; 10 Department of Pathology, The People’s Hospital of Lu’an, Lu’an, China; 11 Quanticision Diagnostics, Inc., Chapel Hill, NC, United States; 12 L.Chambers Biomedical/Biotechnology Research Institute and Department of Biological & Biomedical Sciences, North Carolina Central University, Durham, NC, United States; 13 Department of Breast Surgery, Ruijin Hospital, Shanghai Jiaotong University School of Medicine, Shanghai, China

**Keywords:** CDx, ErbB2, HER2-low, QDB, quantitative, trastuzumab deruxtecan

## Abstract

**Purpose:**

Current daily usage of Trastuzumab Deruxtecan (T-DXd) is guided by immunohistochemistry (IHC)-based HER2 assessment, with known inconsistency and inaccuracy to differentiating IHC 0 from 1 +. In this study, a quantitative HER2 assay based on the Quantitative Dot Blot (QDB) method was explored to fill this unmet need.

**Methods:**

Consecutive resection specimens of HER2 IHC 0 and 1+ from invasive breast cancer patients were assigned to training (n=106) and validation cohorts (*n=*119), respectively by admission time. Protein lysates were extracted from 2x5 μm FFPE slices for HER2 quantification while the adjacent slice was used for IHC staining.

**Results:**

QDB was demonstrated to be more consistent than IHC with an inter-rater Intraclass Correlation Coefficient (ICC) of 0.877 (95%CI: 0.840-0.908) vs. 0.513 (95%CI: 0.433-0.601). Receiver Operating Characteristic (ROC) analysis was performed benchmarked with unanimous agreement of 18 pathologists in the training cohort to achieve an Area Under the Curve (AUC) of 0.9477 (p<0.005). A cutoff of 0.2746 nmole/g was also identified with its imprecision interval to stratify specimens into 0 (<C_5_), equivocal (≥ C_5_ & ≤ C_95_) and 1+ (> C_95_), with overall concordance of 90.9% and 87.3% when benchmarked with unanimous and consensus agreement (≥75%) of pathologists in the training cohort, and 92.0% and 90.5% when validated double-blinded with 12 pathologists in the validation cohort. More importantly, even among specimens unanimously categorized as IHC 0, there were ~15% specimens classified as 1+ by QDB.

**Conclusion:**

Our results supports the QDB HER2 assay as an alternative option to guide T-DXd daily usage by distinguish Her2-low from Her2 0 while setting the stage for outcome-based patient stratification.

## Introduction

The standard practice of combining immunohistochemistry (IHC) with Fluorescence in Situ Hybridization (FISH) as a companion diagnostic assay (CDx) for HER2/ERBB2 assessment has guided the use of anti-HER2 therapy in daily clinical practice for almost 20 years (1). However, it has become increasingly inadequate with the recent expansion of Trastuzumab Deruxtecan (T-DXd) to HER2-low and ultra-low patients (2–4).

Current standard practice categorizes HER2 levels into 0, 1+, 2+, and 3+ by IHC staining, with IHC 0/1+ as HER2 negative (HER2-) and IHC 3+ as HER2 positive (HER2+). The IHC 2+ group is deemed equivocal, requiring further testing by FISH. We considered this algorithm as a compromise to the **qualitative** nature of IHC assessment. We may use the visible color spectrum to illustrate this point. There is no consensus cutoff between yellow and green without the help of a defined quantitative wavelength (570nm). Likewise, there is no consensus cutoff to separate HER2- from HER2+ by IHC alone. Instead, IHC scores are categorized into HER2- with confidence (0 and 1+), HER2+ with confidence (3+), and equivocal cases (2+) to be assessed through FISH analysis. Thus, the current system successfully dodges the search for this nonexistent cutoff in the IHC system.

However, this strategy faces enormous challenges with HER2-low patients. These are patients defined as IHC 1+ or 2+/ISH not-amplified (5). For these patients, pathologists are asked to find a subtle difference between 0 and 1+ by IHC alone. No wonder a recent study claimed “the agreement for assigning a HER2 IHC score of 0 vs not 0 is only slightly better than a coin flip” (2). The situation becomes even worse with the introduction of “HER2 ultra-low”, defined as “IHC 0 with membrane staining” (3). Ensuring consistent interpretation among pathologists in this group may prove even more difficult than for HER2-low patients in daily practice.

There is a broad consensus that a **quantitative** HER2 assay is the only solution (2, 3, 6–8). Multiple efforts have been launched accordingly. However, IHC is not developed for identifying HER2-low and HER2-ultralow (3), so optimization of IHC scoring through automatic scoring or artificial intelligence (AI) becomes an unlikely choice. Mass spectrometry (MS)-based methods requires large amounts of protein lysates and involve complex analytical processes, making them an unlikely fit for daily practice (9). RT-PCR measures mRNA expression, but both Herceptin and T-DXd target the HER2 protein. The discrepancy between RNA and protein, dictated by the central dogma, would become unavoidable (10).

We previously reported a quantitative HER2 assay using the Quantitative Dot Blot (QDB) method for breast cancer patients. For a quantitative assay, a cutoff is needed to stratify patients for the right treatment. Using the only FDA approved antibody for T-DXd stratification, the PATHWAY anti-HER2/neu (4B5) Rabbit Monoclonal Primary Antibody, a validated cutoff at 0.399 nmole/g was identified when benchmarked with the standard HER2 assessment to stratify patients into HER2+ and HER2-. We achieved overall specificity and sensitivity at 98.22% and 96.58% respectively (n*=*577) (11, 12). This method was further suggested as a potential alternative to identify HER2-low based on preliminary data (11). The current study was a follow-up to test this hypothesis.

Current IHC-based HER2-low definition clearly is in crisis, with a “F” grade performance (59.41% agreement) among pathologists across the US (13). Yet, until this definition is revised by regulatory agencies globally or before the outcomes data are available, we are placed in a validation paradox: we have to benchmark a more objective assay with a more subjective assay.

To fix this issue, we invited a group of experienced pathologists (*n=*18) across China to score the same set of IHC slides and used their unanimous agreement as the benchmark to maximally eliminate potential subjectivity in the assessment. A putative cutoff was developed using Receiver Operating Characteristic (ROC) analysis to convert absolutely quantitated HER2 protein levels into 0 and 1+ in the training cohort. The imprecision interval of the putative cutoff was developed and further validated in a validation cohort (*n=*119) to evaluate its utility in daily clinical practice.

## Materials and methods

### Specimen selection

The inclusion criteria for this retrospective observational study was resection specimens from patient diagnosed with invasive breast cancer with FFPE specimens archived in the Yantai Affiliated Hospital of Binzhou Medical University at Yantai, China from April. 2017 to Oct. 2024. They were further limited to IHC 0 and 1+ based on medical record. The specimen must have more than 50% tumor tissue based on H&E staining with no more than 10% tumor classified as ductal or lobular carcinoma *in situ* (DCIS/LCIS), and fixed in 10% neutral buffered formalin for 6-72 hours as recommended in ASCO/CAP guidelines (5, 14). The study was performed in accordance with the Declaration of Helsinki, and was approved by the medical ethics committee of Yantai Affiliated Hospital of Binzhou Medical University (Approval #20230612028), with informed consent forms waived for archived specimens.

### General reagents

All of the chemicals were purchased from Sinopharm Chemicals (Beijing, P. R. China). Recombinant human HER2 protein was purchased from Sino Biological Inc. (Beijing, P. R. China). Ventana anti-HER2/neu (4B5) rabbit monoclonal primary antibody was purchased from Roche Diagnostics GmbH. HRP labeled Donkey Anti-Rabbit IgG secondary antibody was purchased from Jackson Immunoresearch lab (West Grove, PA, USA). BCA protein quantification kit was purchased from Thermo Fisher Scientific Inc. (Carlsbad, CA, USA). QDB plates were provided by Quanticision Diagnostics, Inc. (RTP, USA).

### IHC analysis

IHC analysis was performed on adjacent FFPE sections of each specimen with the standard streptavidin–biotin complex method with 3, 3’-diaminobenzidine as the chromogen. Staining for HER2 was performed on the BenchMark Ultra staining instrument using manufacture recommended staining procedure along with breast cancer tissue control of 0, 1+, 2+, 3 + (5). HER2 interpretation was performed according to the 2023 ASCO/CAP update (5). All invited pathologists were certified with more than 10 years of experience. All the IHC evaluations were blind to the QDB results, and vice versa, in the study. To mitigate the potential bias, all data were reported to Dr. Hao, the project organizer, to support independent statistical analysis.

### QDB analysis

**Figure 1:**
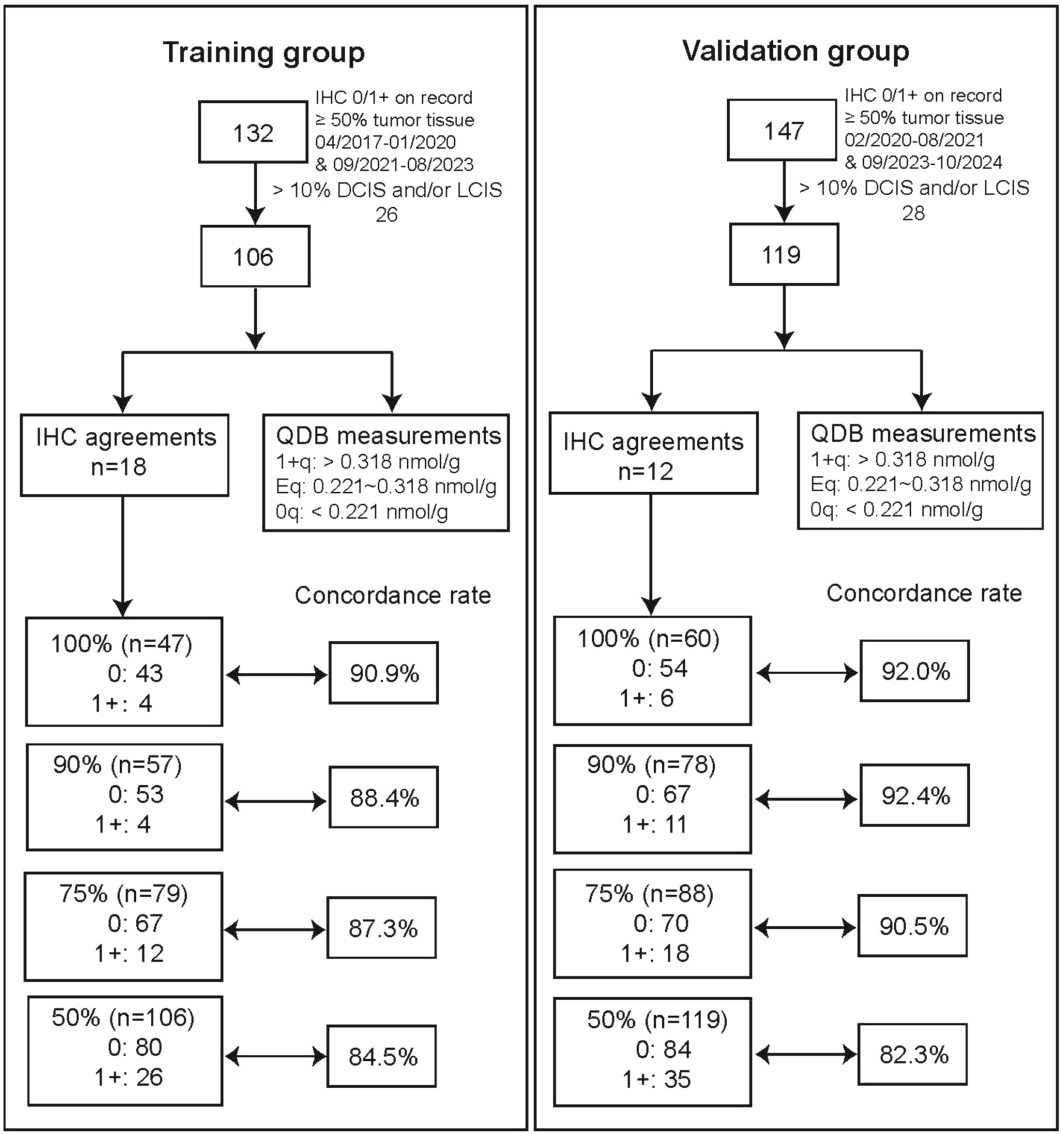
Flow chart of study design and summarization of study results.

QDB analysis as well as lysate preparation were performed as described in previous studies including workflow logistics (11, 12). In brief, two FFPE tissue slices of 5 μm were de-paraffinized and solubilized using lysis buffer (50 mM HEPES, 137 mM NaCl, 5 mM EDTA, 1 mM MgCl_2_, 10 mM Na_2_P_2_O_7_, 1% Triton X-100, and 10% glycerol). The supernatants were collected after centrifugation at 13,800g for 10 min, and the total amount of protein was determined using the BCA protein assay kit (Thermo Fisher Scientific, Waltham, MA). The process of QDB assay was illustrated in detail in **Figure 1** of a previous study (12). The final concentration of FFPE tissue lysates was adjusted to 0.25 μg/μl, and loaded onto the QDB plate at 0.5 μg/unit in triplicate alongside with the protein standard. The plates were dried at room temperature for 4 h before they were blocked in blocking buffer [4% non-fat milk in Tris-buffered saline with 0.1% Tween 20 (TBST)] for another hour. The plates were incubated with 4B5 at 1:10 in PBS overnight at 4 °C. After incubating with an HRP-labeled donkey anti-rabbit secondary antibody for another 4 h at RT, the plates were rinsed with TBST, and washed with TBST 5 × 10 min before they were inserted into a white 96-well plate pre-filled with 100 μl/well ECL working solution for 6 min. The plates were quantified using a Tecan Infinite 200PRO Microplate reader with the option “plate with cover”. FFPE tissues must be prepared by adhering to the ASCO/CAP guidelines on Her2 testing to avoid interference from pre-analytical factors (5). BT-474 and HEK-293 cell lysates with known HER2 level were included in each plate as internal positive and negative controls. Deviation beyond 30% of known HER2 levels of BT-474 was deemed unacceptable.

### Characterization of QDB-based HER2 assay

The limit of detection (LOD) was calculated as Mean + 3SD by measuring 24 replicates of blanks at 0.055, 0.091 and 0.093 nmole/g with average at 0.080 nmole/g. The low limit of quantitation (LLOQ) was calculated as the lowest concentration of HER2 protein standard back calculated within 80~120% of the value with CV ≤ 20% at 0.185 nmol/g. The upper limit of quantitation was defined by the linear range of the protein standard in QDB assay to be >30 nmole/g.

Considering the purpose of this assay is to identify specimens with low HER2 expression, the spike assay was designed by adding 10 pg, 25 pg and 100 pg formalin fixed HER2 protein respectively into protein lysates prepared from FFPE specimens with no HER2 expression, while the control was set by adding dilution buffer to the same lysate. The recovery rate was calculated by subtracting HER2 level of control and compared with the theoretical value. The experiments were repeated three times, with all values within the 20% variations of predicted values (10 pg, 85.5%, 97.2% and 97.3%; 25 pg, 92.2%, 90.6% and 81.6%; and 100 pg, 96.5%, 91.6% and 102.8%).

### Defining the imprecision interval of putative cutoff

The C5, defining the chance for a value below misclassified into Her2 positive would be less than 5%; and C95, defining the chance for a value above misclassified into Her2 negative would be less than 5%, of the assay were developed as the imprecision interval of the assay. The sample was diluted with 0.25 μg/μl IgG free Bovine Serum Albumin (BSA) to set the initial dosage at 0.454 nmole/g. The sample was further diluted serially at 1: 1.2 ratio to the final dosage of 0.378, 0.315, 0.263, 0.219, 0.182, 0.152, 0.127 nmole/g respectively. The prepared samples were loaded to QDB plate as 36-plicates at each dose, and HER2 levels were measured with QDB analysis. The number of samples at each dose with their HER2 values above 0.2746 nmole/g were judged as positive, and the ratios of positive samples were used to calculate C_5_, C_50_ and C_95_ using the probit model of SPSS software at 0.221 nmole/g, 0.269 nmole/g and 0.318 nmole/g respectively. This experiment was repeated three times with highly consistent results. For results falling within the C5 and C95 equivocal interval, where analytical uncertainty is highest, we recommend a multidisciplinary review. In these cases, the QDB result should be integrated with traditional IHC/ISH findings as well as the broader clinical context to guide treatment decisions. This approach ensures that the quantitative data serves as a supplement to, rather than a replacement for, clinical consensus in borderline scenarios.

### Statistical analysis

The performance of QDB method against IHC was evaluated using Receiver Operative Characteristics (ROC) analysis using Graphpad prism. The intraclass correlation analysis was performed using SPSS 26.0. P value <0.05 is considered statistically significant.

## Results

**Figure 1** summarized the study design and results. The effectiveness of QDB vs standard assessment has been reported in two previous papers (11, 12). The current study focused on developing an assay suitable to identify IHC 0 from 1+ by limiting all specimens in this study to IHC 0 or 1+ based on medical records. The patient characteristics of the training cohort of specimens (*n=*106) were reported in **Table 1**.

**Table 1:** Clinicopathological characteristics of breast cancer FFPE samples (n=106) in training cohort by medical record.

Characteristics	Number of cases (%)
Age (y)	
<50	24 (22.6)
≥50	82 (77.4)
Histological grade	
I	5 (4.7)
II	55 (51.9)
III	46 (43.4)
Pathological tumor size, pT	
T1	42 (39.6)
T2	52 (49.0)
T3	5 (4.7)
T4	6 (5.7)
Unknown	1 (1.0)
Pathological lymph node status, pN	
N0	57 (53.8)
N1	24 (22.6)
N2	8 (7.5)
N3	12 (11.3)
Unknown	5 (4.7)
Histological type	
Infiltrating duct carcinoma NOS	91 (85.8)
Invasive lobular carcinoma (ILC)	6 (5.7)
Mucinous carcinoma (MC)	2 (1.9)
others	7 (6.6)
ER (IHC)	
<1%	26 (24.5)
≥1%	80 (75.5)
PR (IHC)	
<1%	29 (27.4)
≥1%	77 (72.6)
Ki67 (IHC)	
<15%	44 (41.5)
≥15%	62 (58.5)
HER2 (IHC)	
0	69 (65.1)
1+	37 (34.9)

**Figure 2:**
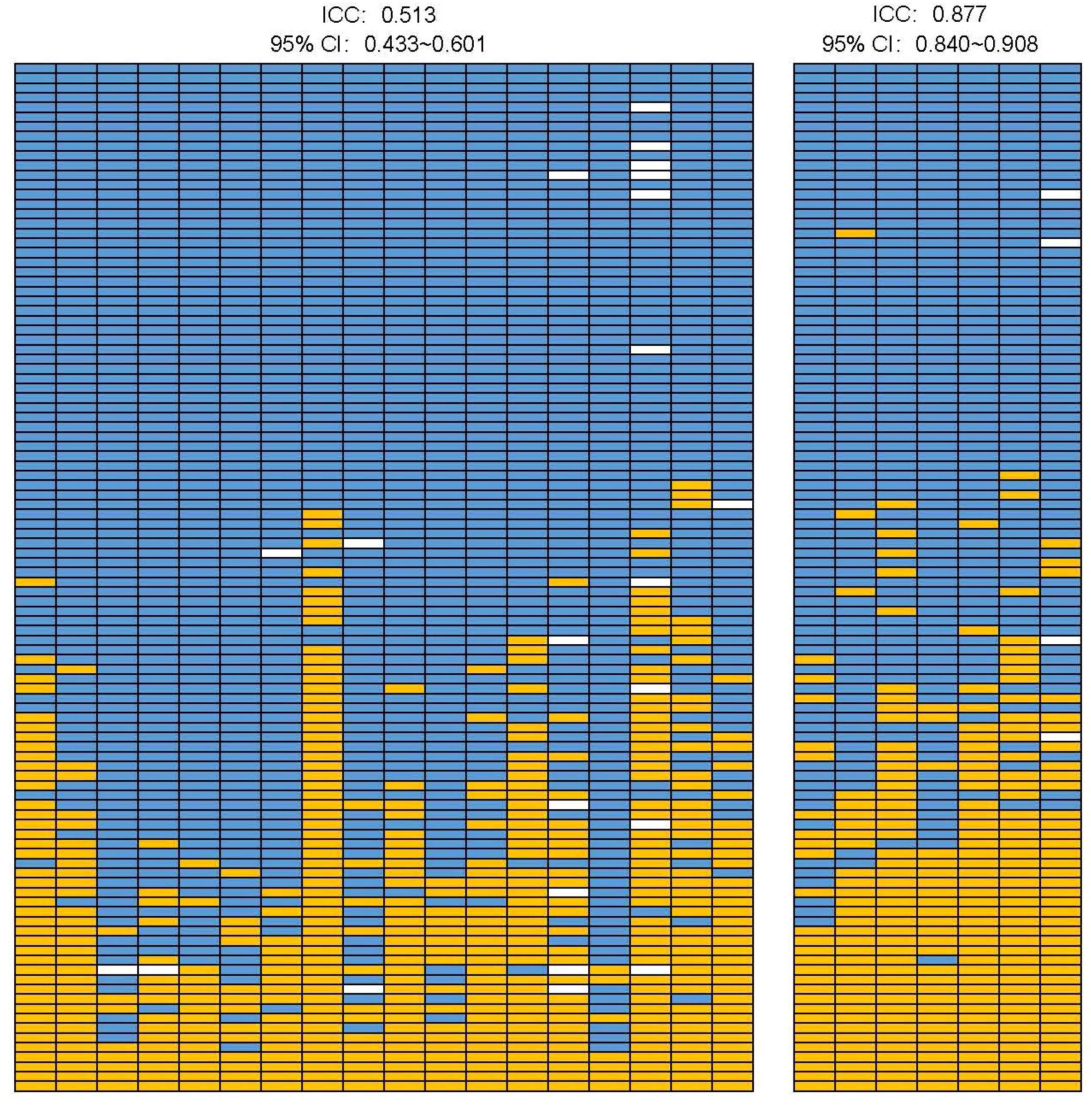
Comparison of the consistency of IHC and QDB method The IHC scores of the training cohort (*n=*106) from 18 pathologists was plotted with IHC 0 in blue and 1+ and above in orange. Those of equivocal cases were in white. The HER2 levels measured with QDB method were also plotted from 7 repeated measurements in a two months periods by two technicians. HER2 levels ≤0.2746 nmole/g was plotted in blue, and >0.2746 nmole/g in orange. The missing values due to insufficient lysates were in white. The inter-rater Intraclass Correlation Coefficient (ICC) was calculated using IHC scores and quantitative HER2 levels using two-way random absolute agreement model.

HER2 protein level in each specimen was repeatedly measured with QDB method 7 times by two technicians spanning two months while the IHC staining was evaluated by 18 experienced pathologists from 9 hospitals across China. The results were reported in **Figure 2** with 1+ for IHC analysis as well as those above the ROC-derived cutoff from QDB measurement in orange, and 0 as well as those below the cutoff in blue, providing a direct comparison of the consistency between the two methods. The detailed data were provided in **Supplementary Tables S1**, **S2** respectively.

The percentage of specimens scored as 1+ in the training cohort varied from 7.55% to 52.83% among 18 pathologists, with a median of 25.47%, and an average of 25.10%. The inter-rater Intraclass Correlation Coefficient analysis (ICC) was calculated as 0.513 (95%CI: 0.433-0.601) using two-way random absolute agreement. There were 43 specimens assigned as IHC 0 and 4 specimens assigned as IHC 1+ unanimously. The number expanded to 53 and 4 with 90% agreement, and 67 and 12 with 75% agreement (consensus) among pathologists. It should be emphasized that there were few specimens maybe categorized as HER2 ultra-low (defined as having less than 10% membrane staining) among the 43 specimens unanimously assigned to IHC 0.

The overall CV of all 7 repeated measurements of HER2 protein levels using the QDB method was averaged at 23.3%. When each measurement was considered as an independent event, the ICC was 0.877 (95%CI: 0.840-0.908), significantly better than that of IHC. (**Figure 2**; **Supplementary Table S2**).

The limit of detection (LOD) was identified at 0.080 nmole/g, the lower limit of quantitation (LLOQ) was at 0.185 nmole/g, and the upper limit of quantification (ULOQ) was > 30 nmole/g. When samples with HER2 protein levels below LLOQ were excluded, the average CV was 17.1%. The spike assay was also performed by adding 10pg, 25 pg and 100 pg HER2 protein into a lysate prepared from a specimen with no HER2 protein expression. The recovery rates were all within the 20% limitation in three independent experiments.

**Figure 3:**
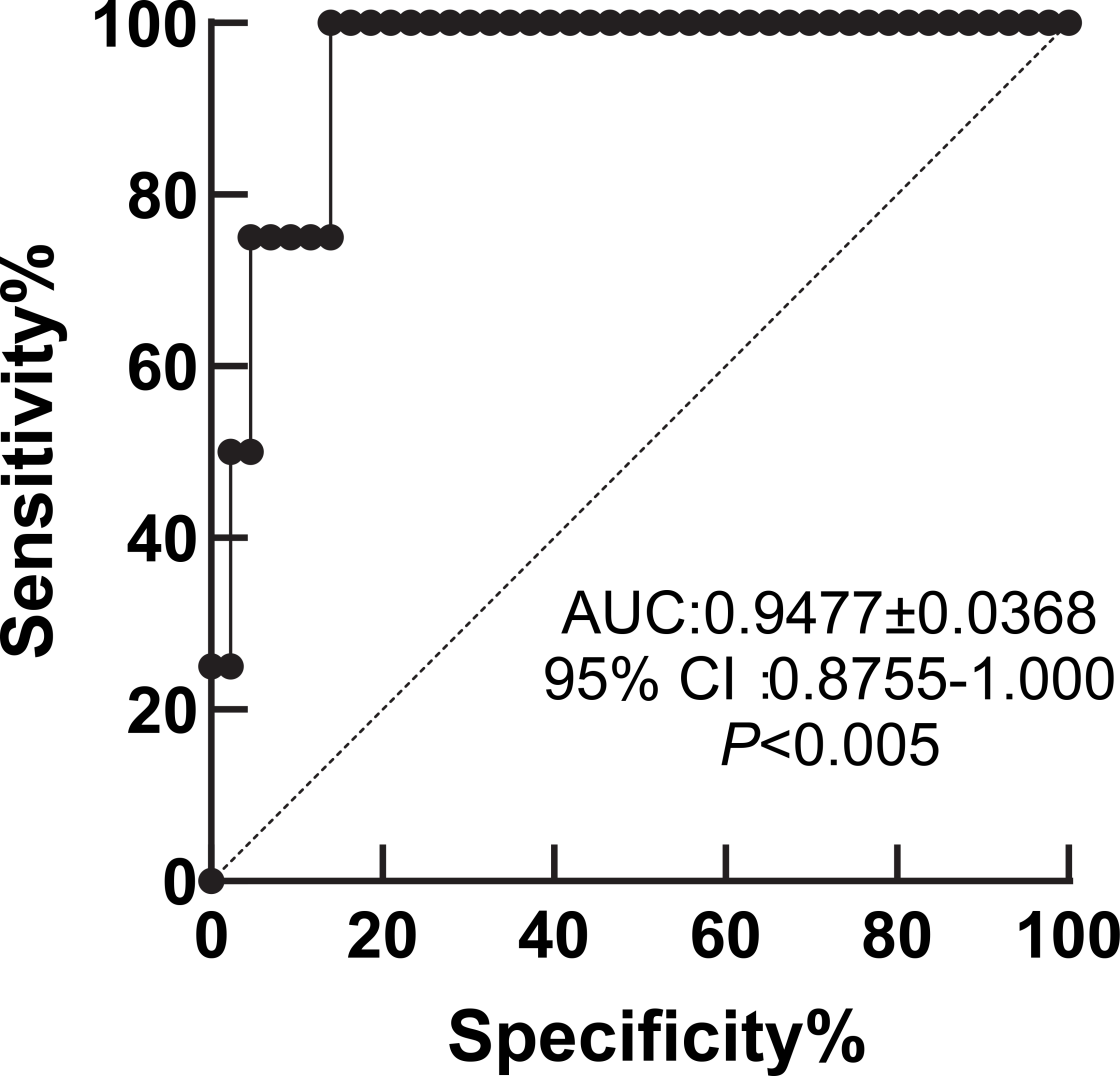
Receiver operative characteristic (ROC) analysis of QDB results benched with IHC scores of unanimous agreement. Specimens with HER2 protein levels measured by QDB method were stratified based on 0 and 1+ by unanimous agreement of 18 pathologists (*n=*47) for ROC analysis. The Area under the curve (AUC) was calculated at 0.9477, with *P* < 0.005.

To evaluate the performance of the QDB HER2 assay against that of IHC, the quantitative results needed to be converted into binary data. Thus, receiver operating characteristics (ROC) analysis was performed using HER2 protein levels of those specimens unanimously classified as IHC 0 and 1+ by all pathologists (*n=*47). We achieved an Area Under the Curve (AUC) of 0.9477, *P* < 0.005 (**Figure 3**). At 0.2746 nmole/g, we achieved sensitivity at 100% (95%CI: 51.01% to 100%), and specificity at 86.05% (95%CI: 72.74% to 93.44%). When all the samples in the training cohort were stratified using this putative cutoff, a total of 36 specimens (34%) were assigned to the 1+ group. Surprisingly, even among specimens unanimously agreed as 0, there was 6 out of 43 (13.4%) specimens identified as 1+ by QDB.

The concept of imprecision interval is routinely used to improve the performance a quantitative assay. The C_5_ (5% chance the result is above 0.2746 nmole/g) and C_95_ (95% chance the result is above 0.2746 nmole/g) of the putative cutoff were identified at 0.221 and 0.318 nmole/g respectively. Accordingly, there were 49 0_q_ (< 0.221 nmole/g), 35 E_q_ for equivocal (between 0.221 and 0.318 nmole/g), and 22 1+_q_ (>0.318 nmole/g) in the training cohort. The concordance between QDB and IHC was calculated at 90.9% among specimens of unanimous agreement (*n=*47), 88.4% among specimens of ≥90% agreement (*n=*57), 87.3% among specimens of consensus or ≥75% agreement (*n=*79), and 84.5% among specimens of ≥50% agreement (*n=*106) (**Supplementary Tables S3a-d**).

This putative cutoff was validated in the validation cohort of 119 breast cancer specimens with the same inclusion criteria. The patient characteristics were reported in **Supplementary Table S4**. Again, FFPE slices were used for QDB measurements and IHC analyses side by side, and the IHC staining was evaluated by 12 experienced pathologists (**Supplementary Tables S5**, **S6**). The percentage of 1+ ranged from 10.9% to 50.4%, with a mean and a median at 27.2% and 26.9%, respectively. There were 54 and 6 specimens assigned as IHC 0 and 1+ unanimously, 70 and 18 specimens by consensus, respectively. The overall ICC was calculated at 0.542 (95%CI: 0.469~0.618).

When specimens were simply stratified by the putative cutoff of 0.2746 nmol/g, the % of 1+ was 22.7%, fairly close to that of IHC. Again, ~13.0% specimens (7 out of 54) were identified as 1+ by QDB among specimens scored as IHC 0 unanimously.

When these specimens were further stratified into 0_q_, E_q_, and 1+_q_, we identified 78 specimens of 0_q,_ 23 of E_q,_ and 18 of 1+_q._ The overall concordance between IHC and QDB in the validation cohort was 92.0% (*n=*60), 92.4% (*n=*78), 90.5% (*n=*88) and 82.3% (*n=*119) by unanimous, ≥90%, consensus and ≥50% among 12 pathologists (**Supplementary Tables S7a-d**).

The overall performance of QDB vs IHC was evaluated by combining training and validation cohorts, and the concordance between QDB and IHC was 91.2% (*n=*107), 90.8% (*n=*135), 89.1% (*n=*167) and 83.2% (*n=*225) when benchmarked by unanimous, 90%, consensus, and 50% agreements among pathologists (**Supplementary Tables S8a-d**).

## Discussion

In this study, we present a quantitative HER2 assay aiming to guide the daily usage of T-DXd by differentiating Her2-low patients from those of IHC 0. The proposed QDB HER2 quantitative assay was extensively evaluated with a validated cutoff and imprecision interval to serve as a more consistent CDx for T-DXd. To the best of our knowledge, this is the only quantitative assay ready to be used in the daily clinical practice. It provides the analytical certainty to circumvent current limitations of IHC to offer more objective assessment of Her2-low for T-Dxd recommendation over average pathologist in daily clinical practice. Yet, the suggested cutoff of 0.2746 nmol/g is aligned well only with current pathological definition with Her-low and possibly become outdated with the availability of T-Dxd efficacy data in the future.

QDB HER2 CDx is a high throughput assay, requiring minimum effort to be adopted in daily clinical practice globally. This assay is capable of processing hundreds to thousands of clinical specimens simultaneously by entry-level technicians, with only a few pathologists required in the central lab to ensure the results are correctly interpreted to meet clinical need of regional areas and beyond.

QDB may be considered the closest thing to IHC among all protein techniques. We expect it to match perfectly with IHC if both are properly performed. However, current IHC staining is optimized for the distinction of 3+ from non-3+ specimens. It might not be sufficient to warrant accurate detection of 1+ specimens. The resulting background would lead to poor discrimination of the weak staining signals. This and other factors would contribute to the ~15% of 1+ specimens being missed even by unanimous agreement of pathologists.

Similar findings have also been reported in studies using mass spectrometry and RT-PCR (9, 10). This oversight may lead to hundreds of thousands of patients each year being mistakenly denied access to T-DXd. This observation thus further questions the validity to use IHC-based method to distinguish HER2-low and HER2-ultralow in daily practice. Unless it can be conclusively demonstrated that the efficacy of T-DXd is entirely independent of HER2 expression—which would fundamentally contradict the premise of targeted therapy—a more reliable alternative must be implemented to prevent ongoing confusion within the pathology community. In this regard, our quantitative QDB HER2 assay provides a feasible alternative to current standard practice.

While the cutoff was developed with only 4 unanimously confirmed 1+ contributing to ROC sensitivity estimates, the extensive validation in this study should be sufficiently justify the validity of this cutoff for routine daily practice. It was tested against a much larger pool of samples where consensus was 90% and 75% in the training set. The fact that the assay remains highly accurate across these large groups demonstrated the cutoff was stable for routine practice. The cutoff was further validated in an independent validation cohort, with the same high performance to validate the clinical utility of this cutoff.

One advantage of absolute values of QDB assays is that we can combine cohorts continuously to fine-tune the cutoff with continuing input of new data to reduce inevitable bias associated with the limited sample size in the training cohort. By combining training and validation cohorts together, we achieved a new “training” cohort ready to be validated in future studies. In this combined “training” cohort, we tested a total 30 1+ by 75% agreements from pathologists to demonstrate the robustness of this cutoff.

In this regard, the proposed cutoff should **not** be considered supported by the pharmacodynamics of T-DXd but rather the analytical limitation to detect the minimum amount of HER2 unanimously agreed by pathologists. In other words, an analytical limit of IHC-based HER2-low but not the therapeutic limit of T-DXd. We believe this assay is the first step toward developing the first ever truly pharmacodynamics-dependent HER2 cutoff once the outcome data become available.

Ultimately, the cutoff of a quantitative QDB HER2 assay should be developed base on drug efficacy with absolute quantitated cutoff value. In this regard, the proposed cutoff at 0.2746 nmol/g should be interpreted as the cutoff best aligned with identifying Her2-low, but not necessary Her2-ultralow, based on latest updated drug label. It may deviate from the ideal cutoff developed based on pharmacodynamics of T-Dxd. However, until the true pharmacodynamics-based cutoff is defined and approved by regulatory agencies globally, clinicians including us must adhere to current definitions to guide T-DXd daily usage. Accordingly, any improvement over current standard practice, even if not ideal, would benefit the patients greatly. The aim of current work thus is to provide clinicians a more objective and consistent assay than the standard practice to fill the gap for now, while paving the road toward developing the first outcome-based quantitative CDx in the field of targeted therapy.

While inherent issue with IHC staining is attributed for the discrepancy between QDB and IHC, the influence of percentage of tumor cellularity may be another factor. QDB measures the overall HER2 levels of tissue lysates, and the results are inevitably lowered with low percentage of the tumor cells in tumor tissue. Thus, caution is warranted to interpret a negative result with specimens of low percentage of cellularity with the QDB method in future analyses. In this regard, microdissection may be considered to expand the applicability of the assay. This practice should eliminates the influence of the percentage of tumor tissues (current set as ≥50%) as well as the percentage of tumor cellularity.

There is still improvement regarding the consistency of the QDB assay due to subtle difference between IHC 0 and 1 +. A standard operational procedure (SOP) was developed with two entry-level technicians performing the assay side by side. An additional analysis is repeated by one technician using the same lysate if there is discrepancy between their results. This practice is expected to significantly improve the consistency of the assay. Nonetheless, the variations in the QDB measurements, like all biochemical assays, are unavoidable. How to address this issue will be another area of discussion in the future.

In summary, an objective and quantitative HER2 protein assay was developed to measure HER2 protein levels in FFPE specimens with unmatched consistency and objectivity, ready to be adopted in daily clinical practice. When benchmarked by the unanimous and consensus agreements of a group of experienced pathologists, we achieved overall concordance at 91.6% and 89.1% respectively. The adoption of this assay in daily clinical practice should liberate pathologists from “guessing” the 0 and 1+ by IHC. More importantly, this assay sets the stage to develop the first ever CDx at protein quantitative level based on the drug efficacy, rather than current qualitative IHC analysis.

## Data Availability

The original contributions presented in the study are included in the article/**Supplementary Material**. Further inquiries can be directed to the corresponding authors.
