# Supplementary material for: Bridging practice and precision: a quantitative HER2 protein assay ready for clinical use in guiding trastuzumab deruxtecan therapy
Source: Front Oncol. 2026 Apr 27;16:1747961. doi: 10.3389/fonc.2026.1747961 (PMC13158065; doi:10.3389/fonc.2026.1747961)
Supplement: Supplementary file 1 [file Table1.docx]

**Supplemental table S1：The results of IHC staining evaluation by 18 pathologists.**

| Specimen ID | Rater 1 | Rater 2 | Rater 3 | Rater 4 | Rater 5 | Rater 6 | Rater 7 | Rater 8 | Rater 9 | Rater 10 | Rater 11 | Rater 12 | Rater 13 | Rater 14 | Rater 15 | Rater 16 | Rater 17 | Rater 18 | Sum* |
| --- | --- | --- | --- | --- | --- | --- | --- | --- | --- | --- | --- | --- | --- | --- | --- | --- | --- | --- | --- |
| 2 | 0 | 0 | 0 | 0 | 0 | 0 | 0 | 0 | 0 | 0 | 0 | 0 | 0 | 0 | 0 | 0 | 0 | 0 | 0 |
| 5 | 0 | 0 | 0 | 0 | 0 | 0 | 0 | 0 | 0 | 0 | 0 | 0 | 0 | 0 | 0 | 0 | 0 | 0 | 0 |
| 14 | 0 | 0 | 0 | 0 | 0 | 0 | 0 | 0 | 0 | 0 | 0 | 0 | 0 | 0 | 0 | 0 | 0 | 0 | 0 |
| 16 | 1 | 0 | 0 | 0 | 0 | 0 | 0 | 1 | 0 | 0 | 0 | 1 | 0 | 1 | 0 | 1 | 0 | 0 | 5 |
| 18 | 0 | 0 | 0 | 0 | 0 | 0 | 0 | 0 | 0 | 0 | 0 | 0 | 0 | 0 | 0 | 0 | 0 | 0 | 0 |
| 27 | 0 | 0 | 0 | 0 | 0 | 0 | 0 | 0 | 0 | 0 | 0 | 0 | 0 | 0 | 0 | E | 0 | 0 | 0 |
| 28 | 1 | 1 | 0 | 0 | 0 | 0 | 0 | 1 | 0 | 1 | 1 | 1 | 1 | 1 | 0 | 1 | 1 | 1 | 11 |
| 30 | 1 | 1 | 0 | 0 | 0 | 0 | 0 | 1 | 0 | 0 | 0 | 0 | 1 | 0 | 0 | 1 | 0 | 1 | 6 |
| 31 | 1 | 1 | 1 | 0 | 1 | 1 | 0 | 2 | 1 | 1 | 1 | 1 | 1 | 1 | 0 | 1 | 1 | 1 | 15 |
| 35 | 0 | 0 | 0 | 0 | 0 | 0 | 0 | 0 | 0 | 0 | 0 | 0 | 0 | 0 | 0 | 0 | 0 | 0 | 0 |
| 37 | 1 | 0 | 0 | 0 | 0 | 0 | 0 | 0 | 0 | 0 | 0 | 0 | 0 | 1 | 0 | E | 0 | 0 | 2 |
| 39 | 0 | 0 | 0 | 0 | 0 | 0 | 0 | 1 | 0 | 0 | 0 | 0 | 1 | 0 | 0 | 1 | 0 | 0 | 4 |
| 41 | 0 | 0 | 0 | 0 | 0 | 0 | 0 | 0 | 0 | 0 | 0 | 0 | 0 | 0 | 0 | 0 | 0 | 0 | 0 |
| 42 | 1 | 1 | 0 | 0 | 0 | 0 | 0 | 1 | 0 | 0 | 0 | 0 | 1 | 0 | 0 | 1 | 1 | 0 | 6 |
| 43 | 0 | 0 | 0 | 0 | 0 | 0 | 0 | 0 | 0 | 0 | 0 | 0 | 0 | 0 | 0 | 0 | 0 | 0 | 0 |
| 48 | 0 | 0 | 0 | 0 | 0 | 0 | 0 | 1 | 0 | 0 | 0 | 0 | 0 | 0 | 0 | 1 | 0 | 0 | 2 |
| 50 | 1 | 1 | 1 | 0 | 0 | 1 | 0 | 2 | 1 | 1 | 1 | 1 | 1 | 1 | 0 | 1 | 1 | 1 | 12 |
| 53 | 0 | 0 | 0 | 0 | 0 | 0 | 0 | 0 | 0 | 0 | 0 | 0 | 0 | 0 | 0 | E | 0 | 0 | 0 |
| 56 | 1 | 0 | 0 | 0 | 0 | 0 | 0 | 1 | 0 | 0 | 0 | 0 | 1 | 0 | 0 | 0 | 1 | 0 | 4 |
| 57 | 1 | 0 | 0 | 0 | 0 | 0 | 0 | 1 | 0 | 0 | 0 | 0 | 1 | 0 | 0 | 1 | 1 | 0 | 5 |
| 59 | 0 | 0 | 0 | 0 | 0 | 0 | 0 | 1 | 0 | 0 | 0 | 0 | 0 | 0 | 0 | 1 | 1 | 0 | 3 |
| 61 | 1 | 1 | 0 | 1 | 0 | 0 | 1 | 1 | 0 | 0 | 1 | 1 | 1 | E | 0 | 1 | 1 | 1 | 11 |
| 66 | 1 | 1 | 0 | 0 | 0 | 0 | 0 | 1 | 0 | 0 | 0 | 1 | 1 | 1 | 0 | E | 1 | 1 | 8 |
| 67 | 1 | 1 | 0 | 1 | 0 | 0 | 1 | 1 | 0 | 1 | 1 | 1 | 1 | 1 | 0 | 1 | 1 | 2 | 13 |
| 69 | 1 | 1 | 1 | 2 | 1 | 1 | 1 | 2 | 1 | 1 | 1 | 1 | 1 | 2 | 1 | 1 | 2 | 1 | 18 |
| 70 | 1 | 0 | 0 | 1 | 1 | 0 | 0 | 1 | 1 | 1 | 0 | 0 | 1 | 1 | 0 | 1 | 2 | 1 | 11 |
| 72 | 0 | 0 | 0 | 0 | 0 | 0 | 0 | 0 | 0 | 0 | 0 | 0 | 0 | 0 | 0 | 0 | 0 | 0 | 0 |
| 74 | 1 | 1 | 0 | 0 | 0 | 0 | 1 | 1 | 0 | 1 | 1 | 1 | 1 | 2 | 0 | 0 | 1 | 1 | 11 |
| 75 | 0 | 0 | 0 | 0 | 0 | 0 | 0 | 0 | 0 | 0 | 0 | 0 | 0 | 0 | 0 | 0 | 1 | 0 | 1 |
| 76 | 0 | 0 | 0 | 0 | 0 | 0 | 0 | 0 | 0 | 0 | 0 | 0 | 0 | 0 | 0 | 0 | 1 | 0 | 1 |
| 77 | 0 | 0 | 0 | 0 | 0 | 0 | 0 | 0 | 0 | 0 | 0 | 0 | 0 | 0 | 0 | 0 | 1 | E | 1 |
| 79 | 0 | 1 | 0 | 0 | 0 | 0 | 0 | 1 | 0 | 0 | 0 | 1 | 0 | 0 | 0 | 1 | 0 | 0 | 4 |
| 80 | 1 | 0 | 0 | 0 | 0 | 0 | 0 | 1 | 0 | 0 | 0 | 0 | 1 | 0 | 0 | 1 | 0 | 1 | 5 |
| 84 | 1 | 1 | 0 | 0 | 0 | 1 | 1 | 1 | 0 | 1 | 1 | 1 | 1 | 0 | 0 | 1 | 1 | 1 | 12 |
| 85 | 1 | 0 | 0 | 0 | 0 | 0 | 0 | 1 | 1 | 1 | 0 | 0 | 1 | E | 0 | 1 | 1 | 0 | 7 |
| 88 | 0 | 0 | 0 | 0 | 0 | 0 | 0 | 0 | 0 | 0 | 0 | 0 | 0 | 0 | 0 | E | 0 | 0 | 0 |
| 91 | 2 | 2 | E | E | 2 | 0 | 2 | 2 | 2 | 2 | 0 | 2 | 0 | E | 2 | E | 2 | 2 | 13 |
| 94 | 1 | 0 | 0 | 0 | 0 | 0 | 0 | 1 | 0 | 0 | 0 | 0 | 0 | 0 | 0 | 1 | 0 | 1 | 4 |
| 95 | 1 | 0 | 0 | 0 | 0 | 0 | 0 | 1 | 0 | 0 | 0 | 0 | 0 | 0 | 0 | 1 | 1 | 1 | 5 |
| 96 | 0 | 0 | 0 | 0 | 0 | 0 | 0 | 0 | 0 | 0 | 0 | 0 | 0 | 0 | 0 | 1 | 1 | 0 | 3 |
| 98 | 1 | 1 | 0 | 1 | 1 | 0 | 1 | 2 | 1 | 1 | 0 | 1 | 1 | 1 | 1 | 1 | 2 | 1 | 15 |
| 100 | 0 | 0 | 0 | 0 | 0 | 0 | 0 | 0 | 0 | 0 | 0 | 0 | 0 | E | 0 | E | 0 | 0 | 0 |
| 102 | 0 | 0 | 0 | 0 | 0 | 0 | 0 | 0 | 0 | 0 | 0 | 0 | 0 | 0 | 0 | 0 | 0 | 0 | 0 |
| 104 | 1 | 0 | 0 | 0 | 0 | 0 | 0 | 1 | 0 | 1 | 0 | 0 | 1 | 0 | 0 | E | 0 | 0 | 4 |
| 106 | 2 | 2 | 2 | 2 | 2 | 1 | 1 | 2 | 2 | 2 | 1 | 1 | 1 | 2 | 2 | 2 | 2 | 2 | 18 |
| 109 | 1 | 1 | 0 | 1 | 1 | 1 | 1 | 1 | 0 | 1 | 1 | 1 | 1 | 1 | 0 | 1 | 2 | 1 | 15 |
| 111 | 0 | 0 | 0 | 0 | 0 | 0 | 0 | 0 | 0 | 0 | 0 | 0 | 1 | E | 0 | 0 | 1 | 0 | 3 |
| 113 | 0 | 0 | 0 | 0 | 0 | 0 | 0 | 0 | 0 | 0 | 0 | 0 | 0 | 0 | 0 | E | 0 | 0 | 0 |
| 114 | 1 | 1 | 0 | 1 | 1 | 0 | 1 | 2 | 0 | 1 | 0 | 1 | 1 | 1 | 1 | 1 | 2 | 1 | 14 |
| 115 | 0 | 0 | 0 | 0 | 0 | 0 | 0 | 0 | 0 | 0 | 0 | 0 | 0 | 0 | 0 | 0 | 0 | 0 | 0 |
| 121 | 1 | 0 | 0 | 0 | 0 | 0 | 0 | 1 | 0 | 1 | 0 | 1 | 1 | 0 | 0 | 0 | 1 | 0 | 6 |
| 122 | 1 | 1 | 0 | 1 | 1 | 1 | 1 | 2 | E | 2 | 1 | 1 | 1 | E | 0 | 1 | 2 | 2 | 14 |
| 123 | 0 | 0 | 0 | 0 | 0 | 0 | 0 | 0 | 0 | 0 | 0 | 0 | 0 | 0 | 0 | 0 | 0 | 0 | 0 |
| 124 | 0 | 0 | 0 | 0 | 0 | 0 | 0 | 1 | 0 | 0 | 0 | 1 | 1 | 1 | 0 | 0 | 0 | 1 | 6 |
| 125 | 0 | 0 | 0 | 0 | 0 | 0 | 0 | 1 | 0 | 0 | 0 | 0 | 0 | 0 | 0 | 0 | 0 | 0 | 1 |
| 126 | 1 | 0 | 0 | 0 | 0 | 0 | 0 | 1 | 0 | 1 | 0 | 0 | 1 | 1 | 0 | 1 | 1 | 1 | 8 |
| 127 | 0 | 0 | 0 | 0 | 0 | 0 | 0 | 0 | 0 | 0 | 0 | 0 | 0 | 0 | 0 | 0 | 0 | 0 | 0 |
| 128 | 0 | 0 | 0 | 0 | 0 | 0 | 0 | 1 | 0 | 0 | 0 | 0 | 0 | 0 | 0 | 1 | 0 | 0 | 2 |
| 129 | 0 | 0 | 0 | 0 | 0 | 0 | 0 | 1 | 0 | 0 | 0 | 0 | 0 | 0 | 0 | 0 | 0 | 0 | 1 |
| 130 | 0 | 0 | 0 | 0 | 0 | 0 | 0 | 0 | 0 | 0 | 0 | 0 | 0 | 0 | 0 | 1 | 0 | 0 | 1 |
| 132 | 0 | 0 | 0 | 0 | 0 | 0 | 0 | 1 | E | 0 | 0 | 0 | 0 | 0 | 0 | 0 | 0 | 0 | 1 |
| 133 | 1 | 0 | 0 | 0 | 0 | 0 | 0 | 1 | 0 | 0 | 0 | 0 | 1 | 1 | 0 | 1 | 0 | 0 | 5 |
| 134 | 0 | 0 | 0 | 0 | 0 | 0 | 0 | 0 | 0 | 0 | 0 | 0 | 0 | 0 | 0 | 0 | 0 | 0 | 0 |
| 135 | 0 | 0 | 0 | 0 | 0 | 0 | 0 | 0 | 0 | 0 | 0 | 0 | 0 | 0 | 0 | 0 | 0 | 0 | 0 |
| 136 | 2 | 2 | 2 | 2 | 3 | 1 | 2 | 3 | 3 | 2 | 2 | 2 | 2 | 2 | 3 | 1 | 2 | 2 | 18 |
| 137 | 1 | 1 | 0 | 0 | 0 | 0 | 0 | 1 | 0 | 1 | 0 | 0 | 1 | 0 | 0 | 1 | 1 | 0 | 7 |
| 138 | 1 | 1 | 0 | 1 | 0 | 1 | 0 | 1 | 0 | 1 | 1 | 1 | 1 | 0 | 0 | 1 | 0 | 1 | 11 |
| 139 | 1 | 1 | 0 | 0 | 0 | 0 | 1 | 2 | 0 | 1 | 1 | 1 | 1 | 1 | 0 | 1 | 2 | 1 | 12 |
| 140 | 0 | 0 | 0 | 0 | 0 | 0 | 0 | 0 | 0 | 0 | 0 | 0 | 0 | 0 | 0 | 0 | 0 | 0 | 0 |
| 141 | 0 | 0 | 0 | 0 | 0 | 0 | 0 | 0 | 0 | 0 | 0 | 0 | 0 | 0 | 0 | 0 | 0 | 0 | 0 |
| 142 | 0 | 0 | 0 | 0 | 0 | 0 | 0 | 0 | 0 | 0 | 0 | 0 | 0 | 0 | 0 | 0 | 0 | 0 | 0 |
| 143 | 0 | 0 | 0 | 0 | 0 | 0 | 0 | 0 | 0 | 0 | 0 | 0 | 0 | 0 | 0 | 0 | 0 | 0 | 0 |
| 144 | 0 | 0 | 0 | 0 | 0 | 0 | 0 | 0 | 0 | 0 | 0 | 0 | 0 | 0 | 0 | 0 | 0 | 0 | 0 |
| 145 | 0 | 0 | 0 | 0 | 0 | 0 | 0 | 0 | 0 | 0 | 0 | 0 | 0 | 0 | 0 | 0 | 0 | 0 | 0 |
| 146 | 0 | 0 | 0 | 0 | 0 | 0 | 0 | 1 | 0 | 0 | 0 | 0 | 0 | 0 | 0 | 1 | 1 | 0 | 4 |
| 147 | 0 | 0 | 0 | 0 | 0 | 0 | 0 | 1 | 0 | 0 | 0 | 0 | 0 | 0 | 0 | 1 | 1 | 0 | 4 |
| 148 | 0 | 0 | 0 | 0 | 0 | 0 | 0 | 0 | 0 | 0 | 0 | 0 | 0 | 0 | 0 | 0 | 0 | 0 | 0 |
| 149 | 0 | 0 | 0 | 0 | 0 | 0 | 0 | 0 | 0 | 0 | 0 | 0 | 0 | 0 | 0 | 0 | 0 | 0 | 0 |
| 150 | 0 | 0 | 0 | 0 | 0 | 0 | 0 | 0 | 0 | 0 | 0 | 0 | 0 | 0 | 0 | 0 | 0 | 0 | 0 |
| 151 | 0 | 0 | 0 | 0 | 0 | 0 | E | 0 | 0 | 0 | 0 | 0 | 0 | 0 | 0 | 1 | 0 | 0 | 1 |
| 153 | 0 | 0 | 0 | 0 | 0 | 0 | 0 | 0 | 0 | 0 | 0 | 0 | 0 | 0 | 0 | 0 | 0 | 0 | 0 |
| 154 | 0 | 0 | 0 | 0 | 0 | 0 | 0 | 0 | 0 | 0 | 0 | 0 | 0 | 0 | 0 | E | 0 | 0 | 0 |
| 155 | 1 | 1 | 1 | 1 | 1 | 1 | 1 | 1 | 0 | 1 | 0 | 1 | 1 | 1 | 0 | 1 | 0 | 1 | 14 |
| 156 | 0 | 0 | 0 | 0 | 0 | 0 | 0 | 0 | 0 | 0 | 0 | 0 | 0 | 0 | 0 | 0 | 0 | 0 | 0 |
| 157 | 0 | 0 | 0 | 0 | 0 | 0 | 0 | 0 | 0 | 0 | 0 | 0 | 0 | 0 | 0 | 0 | 0 | 0 | 1 |
| 158 | 0 | 0 | 0 | 0 | 0 | 0 | 0 | 0 | 0 | 0 | 0 | 0 | 0 | 0 | 0 | 0 | 0 | 0 | 0 |
| 160 | 1 | 1 | 0 | 1 | 0 | 0 | 0 | 1 | 0 | 1 | 0 | 0 | 1 | 1 | 0 | 1 | 0 | 1 | 9 |
| 161 | 1 | 1 | 0 | 1 | 1 | 1 | 1 | 1 | 1 | 1 | 1 | 1 | 1 | 1 | 0 | 1 | 2 | 1 | 16 |
| 162 | 0 | 0 | 0 | 0 | 0 | 0 | 0 | 0 | 0 | 0 | 0 | 0 | 0 | 0 | 0 | 0 | 0 | 0 | 0 |
| 163 | 0 | 0 | 0 | 0 | 0 | 0 | 0 | 0 | 0 | 0 | 0 | 0 | 0 | 0 | 0 | 0 | 0 | 0 | 0 |
| 164 | 1 | 1 | 0 | 0 | 0 | 0 | 0 | 1 | 0 | 1 | 0 | 0 | 1 | 1 | 0 | 1 | 1 | 1 | 9 |
| 165 | 0 | 0 | 0 | 0 | 0 | 0 | 0 | 0 | 0 | 0 | 0 | 0 | 0 | 0 | 0 | 0 | 0 | 0 | 0 |
| 166 | 0 | 0 | 0 | 0 | 0 | 0 | 0 | 0 | 0 | 0 | 0 | 0 | 0 | 0 | 0 | 0 | 0 | 0 | 0 |
| 167 | 0 | 0 | 0 | 0 | 0 | 0 | 0 | 1 | 0 | 0 | 0 | 0 | 0 | 0 | 0 | 0 | 0 | 0 | 1 |
| 168 | 0 | 0 | 0 | 0 | 0 | 0 | 0 | 1 | 0 | 0 | 0 | 0 | 0 | 0 | 0 | 1 | 0 | 0 | 2 |
| 169 | 0 | 0 | 0 | 0 | 0 | 0 | 0 | 0 | 0 | 0 | 0 | 0 | 0 | 0 | 0 | 0 | 0 | 0 | 0 |
| 170 | 1 | 1 | 0 | 0 | 0 | 1 | 0 | 1 | 0 | 1 | 0 | 1 | 1 | 1 | 0 | 1 | 1 | 0 | 10 |
| 171 | 0 | 0 | 0 | 0 | 0 | 0 | 0 | 0 | 0 | 0 | 0 | 0 | 0 | 0 | 0 | 0 | 0 | 0 | 0 |
| 172 | 0 | 1 | 0 | 0 | 1 | 0 | 0 | 1 | 1 | 1 | 0 | 1 | 0 | 0 | 0 | 1 | 0 | 1 | 9 |
| 173 | 0 | 0 | 0 | 0 | 0 | 0 | 0 | 0 | 0 | 0 | 0 | 0 | 0 | 0 | 0 | 0 | 0 | 0 | 0 |
| 174 | 2 | 2 | 1 | 2 | 2 | 1 | 1 | 2 | 2 | 2 | 1 | 1 | 1 | 2 | 2 | 2 | 2 | 2 | 18 |
| 175 | 0 | 0 | 0 | 0 | 0 | 0 | 0 | 0 | 0 | 0 | 0 | 0 | 0 | 0 | 0 | 0 | 0 | 0 | 0 |
| 176 | 0 | 0 | 0 | 0 | 0 | 0 | 0 | 0 | 0 | 0 | 0 | 0 | 0 | 0 | 0 | 0 | 0 | 0 | 0 |
| 177 | 0 | 0 | 0 | 0 | 0 | 0 | 0 | 0 | 0 | 0 | 0 | 0 | 0 | 0 | 0 | 0 | 0 | 0 | 0 |
| 178 | 0 | 0 | 0 | 0 | 0 | 0 | 0 | 0 | 0 | 0 | 0 | 0 | 0 | 0 | 0 | 0 | 0 | 0 | 0 |
| 180 | 1 | 1 | 1 | 1 | 1 | 0 | 1 | 1 | 1 | 1 | 1 | 1 | 1 | 1 | 0 | 1 | 2 | 1 | 16 |

*Sum is the total number of pathologists to score the specimen as 1+ or above.

**Supplemental table S2: The results of QDB detection.**

| **Specimen ID** | **QDB-1** | **QDB-2** | **QDB-3** | **QDB-4** | **QDB-5** | **QDB-6** | **QDB-7** | **Mean** | **CV** |
| --- | --- | --- | --- | --- | --- | --- | --- | --- | --- |
| 2 | 0.191 | 0.122 | 0.146 | 0.116 | 0.126 | 0.143 | 0.054 | 0.128 | 0.320 |
| 5 | 0.139 | 0.155 | 0.137 | 0.171 | 0.084 | 0.124 | 0.122 | 0.133 | 0.209 |
| 14 | 0.194 | 0.195 | 0.384 | 0.301 | 0.262 | 0.288 | 0.280 | 0.272 | 0.241 |
| 16 | 0.181 | 0.097 | 0.191 | 0.167 | 0.160 | 0.210 | 0.233 | 0.177 | 0.245 |
| 18 | 0.146 | 0.094 | 0.237 | 0.228 | 0.172 | 0.175 | 0.192 | 0.178 | 0.275 |
| 27 | 0.230 | 0.218 | 0.146 | 0.123 | 0.202 | 0.123 | 0.175 | 0.174 | 0.257 |
| 28 | 0.264 | 0.303 | 0.439 | 0.269 | 0.383 | 0.136 | 0.246 | 0.291 | 0.337 |
| 30 | 0.203 | 0.262 | 0.319 | 0.293 | 0.318 | 0.200 | 0.336 | 0.276 | 0.203 |
| 31 | 0.229 | 0.288 | 0.338 | 0.283 | 0.371 | 0.348 | 0.353 | 0.316 | 0.161 |
| 35 | 0.198 | 0.248 | 0.346 | 0.243 | 0.317 | 0.308 | 0.286 | 0.278 | 0.184 |
| 37 | 0.297 | 0.199 | 0.330 | 0.220 | 0.327 | 0.249 | 0.306 | 0.275 | 0.191 |
| 39 | 0.187 | 0.157 | 0.215 | 0.117 | 0.257 | 0.135 | 0.155 | 0.175 | 0.279 |
| 41 | 0.197 | 0.282 | 0.012 | 0.064 | 0.145 | 0.010 | 0.169 | 0.126 | 0.809 |
| 42 | 0.219 | 0.369 | 0.202 | 0.188 | 0.135 | 0.164 | 0.207 | 0.212 | 0.353 |
| 43 | 0.178 | 0.243 | 0.282 | 0.164 | 0.232 | 0.192 | 0.306 | 0.228 | 0.234 |
| 48 | 0.102 | 0.136 | 0.049 | 0.006 | 0.115 | 0.043 | 0.052 | 0.072 | 0.644 |
| 50 | 0.412 | 0.511 | 0.454 | 0.243 | 0.414 | 0.477 | 0.462 | 0.425 | 0.205 |
| 53 | 0.175 | 0.218 | 0.323 | 0.173 | 0.263 | 0.185 | 0.225 | 0.223 | 0.244 |
| 56 | 0.289 | 0.177 | 0.335 | 0.154 | 0.244 | 0.310 | 0.293 | 0.257 | 0.268 |
| 57 | 0.182 | 0.117 | 0.084 | 0.022 | 0.146 | 0.061 | 0.104 | 0.102 | 0.521 |
| 59 | 0.237 | 0.194 | 0.141 | 0.170 | 0.159 | 0.289 | 0.187 | 0.197 | 0.257 |
| 61 | 0.233 | 0.169 | 0.360 | 0.185 | 0.410 | 0.246 | 0.182 | 0.255 | 0.369 |
| 66 | 0.215 | 0.177 | 0.357 | 0.183 | 0.172 | 0.170 | 0.166 | 0.206 | 0.333 |
| 67 | 0.248 | 0.249 | 0.271 | 0.260 | 0.328 | 0.275 | 0.280 | 0.273 | 0.100 |
| 69 | 0.506 | 0.518 | 0.586 | 0.531 | 0.595 | 0.522 | 0.506 | 0.538 | 0.069 |
| 70 | 0.279 | 0.292 | 0.297 | 0.270 | 0.316 | 0.299 | 0.298 | 0.293 | 0.051 |
| 72 | 0.133 | 0.169 | 0.154 | 0.107 | 0.087 | 0.131 | 0.133 | 0.131 | 0.210 |
| 74 | 0.276 | 0.273 | 0.233 | 0.193 | 0.196 | 0.320 | 0.202 | 0.242 | 0.202 |
| 75 | 0.079 | 0.027 | 0.096 | 0.051 | 0.042 | 0.169 | 0.062 | 0.075 | 0.629 |
| 76 | 0.183 | 0.155 | 0.103 | 0.131 | 0.122 | 0.128 | 0.115 | 0.134 | 0.201 |
| 77 | 0.112 | 0.095 | 0.094 | 0.093 | 0.099 | 0.115 | 0.123 | 0.104 | 0.116 |
| 79 | 0.246 | 0.252 | 0.232 | 0.219 | 0.249 | 0.226 | 0.242 | 0.238 | 0.054 |
| 80 | 0.508 | 0.615 | 0.664 | 0.614 | 0.662 | 0.584 | 0.624 | 0.610 | 0.087 |
| 84 | 0.512 | 0.561 | 0.563 | 0.480 | 0.558 | 0.501 | 0.452 | 0.518 | 0.085 |
| 85 | 0.337 | 0.500 | 0.590 | 0.497 | 0.490 | 0.486 | 0.367 | 0.467 | 0.185 |
| 88 | 0.171 | 0.199 | 0.213 | 0.230 | 0.219 | 0.403 | NA | 0.239 | 0.347 |
| 91 | 1.089 | 0.975 | 0.668 | 0.538 | 0.713 | 1.007 | 0.656 | 0.807 | 0.263 |
| 94 | 0.231 | 0.124 | 0.098 | 0.105 | 0.089 | 0.113 | 0.160 | 0.132 | 0.377 |
| 95 | 0.276 | 0.269 | 0.311 | 0.292 | 0.318 | 0.382 | 0.282 | 0.304 | 0.126 |
| 96 | 0.285 | 0.257 | 0.267 | 0.228 | 0.179 | 0.305 | 0.247 | 0.253 | 0.162 |
| 98 | 0.267 | 0.234 | 0.273 | 0.184 | 0.252 | 0.280 | 0.208 | 0.243 | 0.147 |
| 100 | 0.251 | 0.305 | 0.314 | 0.258 | 0.265 | 0.379 | 0.258 | 0.290 | 0.159 |
| 102 | 0.201 | 0.228 | 0.117 | 0.108 | 0.120 | 0.104 | 0.156 | 0.148 | 0.333 |
| 104 | 0.228 | 0.194 | 0.236 | 0.154 | 0.279 | 0.226 | 0.217 | 0.219 | 0.176 |
| 106 | 0.278 | 0.211 | 0.357 | 0.177 | 0.300 | 0.352 | 0.254 | 0.276 | 0.245 |
| 109 | 0.334 | 0.332 | 0.419 | 0.374 | 0.461 | 0.378 | 0.332 | 0.376 | 0.132 |
| 111 | 0.733 | 0.511 | 1.269 | 1.025 | 1.046 | 1.025 | 0.906 | 0.931 | 0.264 |
| 113 | 0.236 | 0.242 | 0.375 | 0.343 | 0.361 | 0.293 | 0.316 | 0.309 | 0.179 |
| 114 | 0.177 | 0.184 | 0.209 | 0.156 | 0.218 | 0.205 | 0.184 | 0.190 | 0.114 |
| 115 | 0.186 | 0.133 | 0.159 | 0.135 | 0.157 | 0.136 | 0.143 | 0.150 | 0.127 |
| 121 | 0.190 | 0.139 | 0.178 | 0.165 | 0.169 | 0.302 | 0.161 | 0.186 | 0.286 |
| 122 | 0.320 | 0.395 | 0.384 | 0.356 | 0.380 | 0.525 | 0.315 | 0.382 | 0.184 |
| 123 | 0.388 | 0.385 | 0.795 | 0.652 | 0.613 | 0.640 | 0.589 | 0.580 | 0.255 |
| 124 | 0.268 | 0.286 | 0.309 | 0.244 | 0.361 | 0.300 | 0.307 | 0.297 | 0.124 |
| 125 | 0.259 | 0.332 | 0.406 | 0.348 | 0.427 | 0.433 | 0.370 | 0.368 | 0.168 |
| 126 | 0.265 | 0.370 | 0.486 | 0.358 | 0.401 | 0.281 | 0.373 | 0.362 | 0.205 |
| 127 | 0.153 | 0.166 | 0.174 | 0.143 | 0.174 | 0.149 | 0.153 | 0.159 | 0.079 |
| 128 | 0.209 | 0.124 | 0.206 | 0.186 | 0.181 | 0.198 | 0.194 | 0.185 | 0.156 |
| 129 | 0.181 | 0.085 | 0.151 | 0.121 | 0.106 | 0.151 | 0.141 | 0.134 | 0.240 |
| 130 | 0.131 | 0.126 | 0.120 | 0.091 | 0.127 | 0.129 | 0.072 | 0.114 | 0.201 |
| 132 | 0.142 | 0.083 | 0.102 | 0.071 | 0.092 | 0.041 | 0.060 | 0.084 | 0.382 |
| 133 | 0.218 | 0.256 | 0.266 | 0.184 | 0.267 | 0.217 | 0.207 | 0.231 | 0.141 |
| 134 | 0.107 | 0.122 | 0.113 | 0.098 | 0.132 | 0.171 | 0.103 | 0.121 | 0.206 |
| 135 | 0.246 | 0.202 | 0.257 | 0.241 | 0.225 | 0.230 | 0.229 | 0.233 | 0.075 |
| 136 | 0.525 | 0.598 | 0.853 | 0.696 | 0.613 | 0.802 | 0.558 | 0.664 | 0.188 |
| 137 | 0.351 | 0.304 | 0.266 | 0.242 | 0.286 | 0.334 | 0.346 | 0.304 | 0.137 |
| 138 | 0.247 | 0.198 | 0.244 | 0.249 | 0.307 | 0.221 | 0.201 | 0.238 | 0.156 |
| 139 | 0.392 | 0.330 | 0.556 | 0.403 | 0.449 | 0.409 | 0.429 | 0.424 | 0.162 |
| 140 | 0.152 | 0.070 | 0.154 | 0.133 | 0.185 | 0.126 | 0.133 | 0.136 | 0.259 |
| 141 | 0.144 | 0.052 | 0.155 | 0.125 | 0.144 | 0.144 | NA | 0.127 | 0.298 |
| 142 | 0.218 | 0.109 | 0.268 | 0.214 | 0.261 | 0.218 | 0.291 | 0.226 | 0.263 |
| 143 | 0.190 | 0.120 | 0.153 | 0.122 | 0.181 | 0.123 | 0.201 | 0.156 | 0.224 |
| 144 | 0.158 | 0.092 | 0.091 | 0.087 | 0.083 | 0.122 | 0.182 | 0.116 | 0.338 |
| 145 | 0.250 | 0.187 | 0.220 | 0.240 | 0.236 | 0.319 | 0.239 | 0.242 | 0.165 |
| 146 | 0.340 | 0.346 | 0.311 | 0.201 | 0.320 | 0.276 | 0.305 | 0.300 | 0.164 |
| 147 | 0.193 | 0.204 | 0.164 | 0.104 | 0.109 | 0.123 | 0.135 | 0.147 | 0.272 |
| 148 | 0.217 | 0.230 | 0.277 | 0.267 | 0.239 | 0.212 | 0.194 | 0.234 | 0.128 |
| 149 | 0.096 | 0.095 | 0.124 | 0.119 | 0.129 | 0.125 | NA | 0.115 | 0.132 |
| 150 | 0.251 | 0.219 | 0.275 | 0.283 | 0.280 | 0.263 | 0.246 | 0.259 | 0.088 |
| 151 | 0.223 | 0.181 | 0.280 | 0.241 | 0.201 | 0.187 | 0.227 | 0.220 | 0.156 |
| 153 | 0.205 | 0.084 | 0.114 | 0.202 | 0.068 | 0.168 | 0.117 | 0.137 | 0.402 |
| 154 | 0.126 | 0.102 | 0.125 | 0.095 | 0.124 | 0.130 | 0.142 | 0.121 | 0.135 |
| 155 | 0.393 | 0.503 | 0.524 | 0.489 | 0.467 | 0.427 | 0.380 | 0.455 | 0.123 |
| 156 | 0.097 | 0.094 | 0.136 | 0.117 | 0.130 | 0.104 | 0.111 | 0.113 | 0.143 |
| 157 | 0.122 | 0.163 | 0.221 | 0.192 | 0.183 | 0.131 | 0.180 | 0.170 | 0.204 |
| 158 | 0.082 | 0.115 | 0.132 | 0.125 | 0.123 | 0.095 | 0.126 | 0.114 | 0.162 |
| 160 | 0.264 | 0.337 | 0.392 | 0.334 | 0.353 | 0.317 | 0.366 | 0.337 | 0.120 |
| 161 | 0.209 | 0.167 | 0.236 | 0.209 | 0.252 | 0.180 | 0.300 | 0.222 | 0.204 |
| 162 | 0.166 | 0.079 | 0.073 | 0.072 | 0.093 | 0.056 | 0.090 | 0.090 | 0.400 |
| 163 | 0.132 | 0.062 | 0.257 | 0.127 | 0.201 | 0.210 | 0.131 | 0.160 | 0.410 |
| 164 | 0.358 | 0.411 | 0.526 | 0.374 | 0.475 | 0.577 | 0.548 | 0.467 | 0.188 |
| 165 | 0.068 | 0.010 | 0.087 | 0.027 | 0.084 | 0.120 | 0.093 | 0.070 | 0.553 |
| 166 | 0.167 | 0.266 | 0.264 | 0.245 | 0.282 | 0.417 | NA | 0.273 | 0.296 |
| 167 | 0.292 | 0.276 | 0.410 | 0.341 | 0.373 | 0.394 | 0.421 | 0.358 | 0.159 |
| 168 | 0.210 | 0.251 | 0.297 | 0.242 | 0.293 | 0.309 | 0.375 | 0.282 | 0.191 |
| 169 | 0.328 | 0.409 | 0.545 | 0.438 | 0.556 | 0.505 | 0.552 | 0.476 | 0.183 |
| 170 | 0.368 | 0.362 | 0.500 | 0.327 | 0.445 | 0.393 | 0.582 | 0.425 | 0.212 |
| 171 | 0.075 | 0.100 | 0.089 | 0.008 | 0.116 | 0.138 | 0.126 | 0.093 | 0.466 |
| 172 | 0.105 | 0.188 | 0.162 | 0.083 | 0.146 | 0.155 | 0.183 | 0.146 | 0.267 |
| 173 | 0.272 | 0.312 | 0.426 | 0.321 | 0.393 | 0.396 | 0.404 | 0.360 | 0.162 |
| 174 | 0.307 | 0.512 | 0.478 | 0.528 | 0.441 | 0.496 | 0.495 | 0.465 | 0.162 |
| 175 | 0.097 | 0.172 | 0.126 | 0.145 | 0.194 | 0.094 | 0.194 | 0.146 | 0.291 |
| 176 | 0.076 | 0.093 | 0.111 | 0.090 | 0.123 | 0.039 | 0.126 | 0.094 | 0.321 |
| 177 | 0.077 | 0.070 | 0.056 | 0.043 | 0.081 | 0.010 | 0.056 | 0.056 | 0.435 |
| 178 | 0.187 | 0.166 | 0.143 | 0.128 | 0.142 | 0.127 | 0.165 | 0.151 | 0.148 |
| 180 | 0.246 | 0.283 | 0.200 | 0.107 | 0.246 | 0.320 | 0.220 | 0.232 | 0.293 |
| average CV |  |  |  |  |  |  |  |  | 0.233 |

**Supplemental table S3a**: Concordance between QDB and IHC for specimens of unanimous agreement (n=47). The concordance rate was calculated at (27+3)/(27+3+3+0)=90.9%.

|  | IHC 0 | IHC 1+ |
| --- | --- | --- |
| 0_q_ | 27 | 0 |
| E_q_ | 13 | 1 |
| 1+_q_ | 3 | 3 |

**Supplemental table S3b:** Concordance between QDB and IHC for specimens of ≥90% agreement (n=57). The concordance rate was calculated at (35+3)/(35+3+5+0)=88.4%.

|  | IHC 0 | IHC 1+ |
| --- | --- | --- |
| 0_q_ | 35 | 0 |
| E_q_ | 13 | 1 |
| 1+_q_ | 5 | 3 |

**Supplemental table S3c**: Concordance between QDB and IHC for specimens of consensus agreement (≥75%) (n=79). The concordance rate was calculated at (42+6)/(42+6+6+1)=87.3%.

|  | IHC 0 | IHC 1+ |
| --- | --- | --- |
| 0_q_ | 42 | 1 |
| E_q_ | 19 | 5 |
| 1+_q_ | 6 | 6 |

**Supplemental table S3d**: Concordance between QDB and IHC for specimens of ≥50% agreement (n=106). The concordance rate was calculated at (47+13)/(47+13+9+2)=84.5%.

|  | IHC 0 | IHC 1+ |
| --- | --- | --- |
| 0_q_ | 47 | 2 |
| E_q_ | 24 | 11 |
| 1+_q_ | 9 | 13 |

**Supplemental table S4. Clinicopathological Characteristics of Breast Cancer FFPE Samples (n=108*) in validation cohort by medical record.**

| **Characteristics** | **Number of Cases (%)** |
| --- | --- |
| **Age (y)** |  |
| <50 | 32 (29.6) |
| ≥50 | 76 (70.4) |
| **Histological grade** |  |
| I | 10 (9.3) |
| II | 59 (54.6) |
| III | 39 (36.1) |
| **Pathological tumor size, pT** |  |
| T1 | 44 (40.7) |
| T2 | 52 (48.1) |
| T3 | 8 (7.4) |
| T4 | 0 (0.0) |
| Unknown | 4 (3.7) |
| **Pathological lymph node status，pN** |  |
| N0 | 62 (57.4) |
| N1 | 27 (25.0) |
| N2 | 10 (9.3) |
| N3 | 8 (7.4) |
| Unknown | 1 (0.9) |
| **Histological type** |  |
| Ductal | 77 (71.3) |
| Lobular | 4 (3.7) |
| others | 27 (25.0) |
| **ER (IHC)** |  |
| <1% | 26 (24.1) |
| ≥1% | 82 (75.9) |
| **PR (IHC)** |  |
| <1% | 38 (35.2) |
| ≥1% | 70 (64.8) |
| **Ki67 (IHC)** |  |
| <15% | 50 (46.2) |
| ≥15% | 57 (52.8) |
| Unknown | 1 (0.9) |
| **HER2 (IHC)** |  |
| 0 | 48 (44.4) |
| 1+ | 60 (55.6) |

*: including 11 additional resection specimens from 10 patients to make total of 119 specimens.

**Supplemental table S5: The results of IHC staining evaluation by 12 pathologists.**

| Specimen ID | Rater 1 | Rater 2 | Rater 3 | Rater 4 | Rater 5 | Rater 6 | Rater 7 | Rater 8 | Rater 9 | Rater 10 | Rater 11 | Rater 12 | Sum* |
| --- | --- | --- | --- | --- | --- | --- | --- | --- | --- | --- | --- | --- | --- |
| 1 | 0 | 0 | 0 | 0 | 0 | 0 | 0 | 0 | 0 | 0 | 0 | 0 | 0 |
| 2 | 0 | 0 | 0 | 0 | 0 | 0 | 0 | 0 | 0 | 0 | 0 | 0 | 0 |
| 3 | 3 | 3 | 3 | 2 | 3 | 3 | 3 | 3 | 3 | 3 | 3 | 3 | 12 |
| 4 | 0 | 0 | 0 | 0 | 0 | 0 | 0 | 0 | 0 | 0 | 0 | 0 | 0 |
| 5 | 0 | 0 | 0 | 0 | 0 | 0 | 0 | 0 | 0 | 0 | 0 | 0 | 0 |
| 6 | 1 | 1 | 0 | 0 | 1 | 0 | 0 | 2 | 0 | 1 | 1 | 1 | 7 |
| 7 | 0 | 0 | 0 | 0 | 0 | 0 | 0 | 0 | 0 | 0 | 0 | 0 | 0 |
| 8 | 1 | 1 | 0 | 0 | 0 | 0 | 0 | 1 | 0 | 1 | 1 | 0 | 5 |
| 9 | 1 | 2 | 1 | 1 | 1 | 1 | 1 | 2 | 1 | 2 | 1 | 1 | 12 |
| 10 | 0 | 0 | 0 | 0 | 0 | 0 | 0 | 0 | 0 | 0 | 0 | 0 | 0 |
| 12 | 0 | 0 | 0 | 0 | 0 | 0 | 0 | 0 | 0 | 0 | 0 | 0 | 0 |
| 13 | 0 | 0 | 0 | 0 | 0 | 0 | 0 | 0 | 0 | 0 | 0 | 0 | 0 |
| 14 | 0 | 0 | 0 | 0 | 0 | 0 | 0 | 0 | 0 | 0 | 0 | 0 | 0 |
| 16 | 1 | 0 | 2 | 1 | 0 | 2 | 0 | 3 | 3 | 3 | 3 | 3 | 9 |
| 18 | 1 | 1 | 0 | 0 | 1 | 1 | 0 | 1 | 0 | 0 | 0 | 0 | 5 |
| 19 | 0 | 0 | 0 | 0 | 0 | 0 | 0 | 0 | 0 | 0 | 0 | 0 | 0 |
| 20 | 0 | 0 | 0 | 0 | 0 | 0 | 0 | 0 | 0 | 0 | 0 | 0 | 0 |
| 21 | 0 | 0 | 0 | 0 | 0 | 0 | 0 | 0 | 0 | 0 | 0 | 0 | 0 |
| 22 | 0 | 1 | 0 | 0 | 0 | 0 | 0 | 1 | 0 | 0 | 0 | 0 | 2 |
| 23 | 0 | 0 | 0 | 0 | 0 | 0 | 0 | 1 | 0 | 0 | 1 | 0 | 2 |
| 25 | 0 | 0 | 0 | 0 | 0 | 0 | 0 | 0 | 0 | 0 | 0 | 0 | 0 |
| 26 | 0 | 0 | 0 | 0 | 0 | 0 | 0 | 0 | 0 | 0 | 0 | 0 | 0 |
| 27 | 0 | 0 | 0 | 0 | 0 | 0 | 0 | 0 | 0 | 0 | 0 | 0 | 0 |
| 28 | 0 | 0 | 0 | 0 | 0 | 0 | 0 | 0 | 0 | 0 | 0 | 0 | 0 |
| 29 | 0 | 0 | 0 | 0 | 0 | 0 | 0 | 1 | 0 | 1 | 1 | 0 | 3 |
| 30 | 0 | 0 | 0 | 0 | 0 | 0 | 0 | 0 | 0 | 0 | 0 | 0 | 0 |
| 31 | 0 | 0 | 0 | 0 | 0 | 0 | 0 | 0 | 0 | 0 | 0 | 0 | 0 |
| 32 | 0 | 0 | 0 | 0 | 0 | 0 | 0 | 0 | 0 | 0 | 0 | 0 | 0 |
| 33 | 0 | 0 | 0 | 0 | 0 | 0 | 0 | 1 | 0 | 0 | 1 | 0 | 2 |
| 34 | 0 | 0 | 0 | 0 | 0 | 0 | 0 | 0 | 0 | 0 | 0 | 0 | 0 |
| 35 | 0 | 0 | 0 | 0 | 0 | 0 | 0 | 0 | 0 | 0 | 0 | 0 | 0 |
| 36 | 1 | 1 | 1 | 0 | 1 | 1 | 1 | 2 | 0 | 2 | 1 | 1 | 10 |
| 37 | 0 | 0 | 0 | 0 | 0 | 0 | 0 | 0 | 0 | 0 | 0 | 0 | 0 |
| 38 | 0 | 0 | 0 | 0 | 0 | 0 | 0 | 1 | 0 | 0 | 0 | 0 | 1 |
| 39 | 0 | 0 | 0 | 0 | 0 | 0 | 0 | 0 | 0 | 0 | 0 | 0 | 0 |
| 40 | 0 | 0 | 0 | 0 | 0 | 0 | 0 | 0 | 0 | 0 | 0 | 0 | 0 |
| 41 | 0 | 0 | 0 | 0 | 0 | 0 | 0 | 0 | 0 | 0 | 0 | 0 | 0 |
| 43 | 0 | 0 | 0 | 0 | 0 | 0 | 0 | 0 | 0 | 0 | 0 | 0 | 0 |
| 44 | 2 | 2 | 2 | 2 | 2 | 1 | 1 | 2 | 1 | 2 | 2 | 2 | 12 |
| 45 | 0 | 0 | 0 | 0 | 0 | 0 | 0 | 1 | 0 | 0 | 0 | 0 | 1 |
| 47 | 0 | 0 | 0 | 0 | 0 | 0 | 0 | 0 | 0 | 0 | 0 | 0 | 0 |
| 49 | 0 | 0 | 0 | 0 | 0 | 0 | 0 | 1 | 0 | 0 | 0 | 0 | 1 |
| 50 | 0 | 1 | 0 | 0 | 0 | 1 | 0 | 1 | 0 | 1 | 0 | 1 | 5 |
| 51 | 1 | 1 | 0 | 0 | 1 | 1 | 1 | 1 | 0 | 0 | 1 | 1 | 8 |
| 52 | 0 | 0 | 0 | 0 | 0 | 0 | 1 | 1 | 0 | 1 | 1 | 1 | 5 |
| 54 | 0 | 0 | 0 | 0 | 0 | 0 | 0 | 1 | 0 | 0 | 0 | 0 | 1 |
| 55 | 0 | 0 | 0 | 0 | 1 | 0 | 0 | 0 | 0 | 0 | 0 | 0 | 1 |
| 56 | 0 | 0 | 0 | 0 | 0 | 0 | 0 | 0 | 0 | 0 | 0 | 0 | 0 |
| 58 | 0 | 0 | 0 | 0 | 0 | 0 | 0 | 0 | 0 | 0 | 0 | 0 | 0 |
| 59 | 1 | 0 | 0 | 0 | 1 | 1 | 0 | 1 | 0 | 0 | 1 | 1 | 6 |
| 60 | 0 | 0 | 0 | 0 | 0 | 0 | 0 | 0 | 0 | 0 | 0 | 0 | 0 |
| 61 | 1 | 0 | 0 | 0 | 0 | 0 | 1 | 0 | 0 | 0 | 1 | 0 | 3 |
| 62 | 0 | 0 | 0 | 0 | 0 | 0 | 0 | 1 | 0 | 0 | 0 | 0 | 1 |
| 63 | 0 | 0 | 0 | 0 | 0 | 0 | 0 | 0 | 0 | 0 | 0 | 0 | 0 |
| 64 | 1 | 0 | 0 | 0 | 0 | 0 | 0 | 2 | 2 | 2 | 1 | 2 | 6 |
| 65 | 0 | 0 | 0 | 0 | 0 | 0 | 0 | 0 | 0 | 0 | 0 | 0 | 0 |
| 66 | 0 | 0 | 0 | 0 | 0 | 1 | 0 | 2 | 0 | 2 | 1 | 2 | 5 |
| 67 | 0 | 1 | 1 | 0 | 0 | 0 | 1 | 1 | 0 | 1 | 1 | 1 | 7 |
| 68 | 0 | 0 | 0 | 0 | 0 | 0 | 0 | 1 | 0 | 0 | 0 | 0 | 1 |
| 69 | 0 | 0 | 0 | 0 | 0 | 0 | 0 | 0 | 0 | 0 | 0 | 0 | 0 |
| 70 | 1 | 1 | 0 | 0 | 0 | 0 | 0 | 1 | 0 | 1 | 1 | 1 | 6 |
| 71 | 1 | 0 | 2 | 0 | 2 | 1 | 2 | 2 | 1 | 2 | 1 | 2 | 10 |
| 73 | 0 | 0 | 0 | 0 | 0 | 0 | 0 | 1 | 0 | 0 | 0 | 0 | 1 |
| 74 | 0 | 0 | 0 | 0 | 0 | 0 | 0 | 0 | 0 | 0 | 0 | 0 | 0 |
| 75 | 1 | 1 | 0 | 0 | 0 | 0 | 0 | 2 | 0 | 2 | 0 | 2 | 5 |
| 76 | 1 | 0 | 0 | 0 | 0 | 0 | 0 | 1 | 0 | 1 | 0 | 1 | 4 |
| 77 | 0 | 0 | 0 | 0 | 0 | 0 | 0 | 0 | 0 | 0 | 0 | 0 | 0 |
| 79 | 0 | 0 | 0 | 0 | 0 | 0 | 0 | 0 | 0 | 0 | 0 | 0 | 0 |
| 80 | 1 | 1 | 0 | 0 | 1 | 0 | 0 | 1 | 1 | 1 | 0 | 1 | 7 |
| 81 | 0 | 0 | 0 | 0 | 1 | 0 | 0 | 0 | 0 | 0 | 0 | 0 | 1 |
| 82 | 0 | 0 | 0 | 0 | 0 | 0 | 0 | 0 | 0 | 0 | 0 | 0 | 0 |
| 83 | 0 | 0 | 0 | 0 | 0 | 0 | 0 | 0 | 0 | 0 | 0 | 0 | 0 |
| 84 | 0 | 0 | 0 | 0 | 0 | 0 | 0 | 1 | 0 | 0 | 0 | 0 | 1 |
| 86 | 1 | 1 | 1 | 1 | 1 | 0 | 1 | 2 | 0 | 2 | 1 | 2 | 10 |
| 87 | 0 | 0 | 0 | 0 | 0 | 0 | 0 | 0 | 0 | 0 | 0 | 0 | 0 |
| 88 | 1 | 1 | 1 | 1 | 1 | 0 | 1 | 2 | 0 | 2 | 0 | 2 | 9 |
| 91 | 0 | 0 | 0 | 0 | 0 | 0 | 0 | 0 | 0 | 0 | 0 | 0 | 0 |
| 92 | 0 | 0 | 0 | 0 | 0 | 0 | 0 | 1 | 0 | 0 | 0 | 0 | 1 |
| 93 | 0 | 0 | 0 | 0 | 0 | 0 | 0 | 0 | 0 | 0 | 0 | 0 | 0 |
| 94 | 0 | 0 | 0 | 0 | 0 | 0 | 0 | 0 | 0 | 0 | 0 | 0 | 0 |
| 95 | 0 | 0 | 0 | 0 | 0 | 0 | 0 | 0 | 0 | 0 | 0 | 0 | 0 |
| 96 | 0 | 0 | 0 | 0 | 0 | 0 | 1 | 0 | 0 | 0 | 0 | 0 | 1 |
| 98 | 0 | 0 | 0 | 0 | 0 | 0 | 0 | 0 | 0 | 0 | 0 | 0 | 0 |
| 99 | 0 | 0 | 0 | 0 | 0 | 0 | 0 | 0 | 0 | 0 | 0 | 0 | 0 |
| 100 | 0 | 0 | 0 | 0 | 0 | 0 | 0 | 0 | 0 | 0 | 0 | 0 | 0 |
| 102 | 0 | 0 | 0 | 0 | 0 | 0 | 0 | 0 | 0 | 0 | 0 | 0 | 0 |
| 103 | 0 | 0 | 0 | 0 | 0 | 0 | 0 | 0 | 0 | 0 | 0 | 0 | 0 |
| 104 | 0 | 0 | 0 | 0 | 0 | 0 | 0 | 0 | 0 | 0 | 0 | 0 | 0 |
| 105 | 1 | 1 | 0 | 0 | 1 | 0 | 1 | 1 | 0 | 1 | 0 | 0 | 6 |
| 106 | 0 | 0 | 0 | 0 | 0 | 0 | 0 | 0 | 0 | 0 | 0 | 0 | 0 |
| 107 | 0 | 0 | 0 | 1 | 0 | 0 | 0 | 1 | 0 | 1 | 0 | 0 | 3 |
| 108 | 1 | 1 | 0 | 1 | 1 | 0 | 1 | 1 | 0 | 1 | 1 | 1 | 9 |
| 110 | 1 | 0 | 0 | 0 | 1 | 1 | 1 | 1 | 0 | 1 | 1 | 1 | 8 |
| 111 | 0 | 0 | 0 | 0 | 0 | 0 | 0 | 0 | 0 | 0 | 0 | 0 | 0 |
| 112 | 1 | 0 | 1 | 0 | 0 | 0 | 0 | 1 | 0 | 1 | 1 | 1 | 6 |
| 114 | 0 | 1 | 0 | 0 | 0 | 1 | 1 | 1 | 1 | 1 | 1 | 1 | 8 |
| 115 | 1 | 0 | 0 | 0 | 0 | 0 | 0 | 1 | 0 | 1 | 0 | 0 | 3 |
| 117 | 1-2 | 1 | 1 | 1 | 1 | 1 | 2 | 2 | 1 | 2 | 1 | 1 | 12 |
| 120 | 1 | 1 | 1 | 1 | 1 | 1 | 1 | 2 | 1 | 2 | 1 | 2 | 12 |
| 121 | 2 | 2 | 1 | 0 | 1 | 1 | 1 | 2 | 1 | 2 | 2 | 1 | 11 |
| 122 | 1 | 0 | 0 | 0 | 0 | 0 | 0 | 1 | 0 | 1 | 0 | 0 | 3 |
| 123 | 1 | 1 | 1 | 0 | 0 | 0 | 1 | 1 | 0 | 1 | 1 | 1 | 8 |
| 124 | 1 | 2 | 1 | 1 | 1 | 1 | 1 | 2 | 1 | 2 | 1 | 1 | 12 |
| 125 | 1 | 1 | 1 | 0 | 0 | 0 | 1 | 1 | 0 | 1 | 1 | 1 | 8 |
| 126 | 0 | 0 | 0 | 0 | 0 | 0 | 0 | 0 | 0 | 0 | 0 | 0 | 0 |
| 127 | 0 | 0 | 1 | 0 | 0 | 0 | 0 | 0 | 0 | 0 | 0 | 0 | 1 |
| 128 | 1 | 1 | 1 | 0 | 1 | 1 | 1 | 1 | 0 | 1 | 1 | 1 | 10 |
| 129 | 2 | 2 | 1 | 1 | 1 | 1 | 1 | 2 | 0 | 2 | 1 | 1 | 11 |
| 130 | 1 | 0 | 0 | 0 | 0 | 0 | 1 | 1 | 0 | 1 | 1 | 1 | 6 |
| 131 | 1 | 0 | 0 | 0 | 0 | 0 | 0 | 1 | 0 | 1 | 0 | 0 | 3 |
| 132 | 1 | 0 | 0 | 0 | 1 | 0 | 1 | 1 | 0 | 1 | 0 | 0 | 5 |
| 133 | 1 | 1 | 1 | 0 | 1 | 0 | 1 | 1 | 0 | 1 | 0 | 1 | 8 |
| 134 | 0 | 0 | 0 | 0 | 0 | 0 | 0 | 0 | 0 | 0 | 0 | 0 | 0 |
| 135 | 0 | 0 | 0 | 0 | 0 | 0 | 0 | 0 | 0 | 0 | 0 | 0 | 0 |
| 136 | 1 | 1 | 0 | 0 | 1 | 1 | 1 | 1 | 0 | 1 | 0 | 0 | 7 |
| 137 | 1 | 1 | 1 | 0 | 1 | 1 | 1 | 2 | 1 | 2 | 1 | 1 | 11 |
| 138 | 1 | 1 | 1 | 0 | 1 | 1 | 1 | 1 | 1 | 1 | 1 | 1 | 11 |
| 139 | 1 | 2 | 0 | 0 | 1 | 0 | 0 | 1 | 0 | 1 | 1 | 1 | 7 |
| 140 | 1 | 1 | 1 | 1 | 1 | 1 | 1 | 2 | 0 | 2 | 1 | 1 | 11 |

*: Sum is the total number of pathologists to score the specimen as 1+ or above.

**Supplemental table S6：The results of QDB detection.**

| Specimen ID | QDB1 | QDB2 | mean | CV |
| --- | --- | --- | --- | --- |
| 1 | 0.192 | 0.202 | 0.197 | 0.037 |
| 2 | 0.197 | 0.150 | 0.173 | 0.195 |
| 3 | 5.418 | 4.526 | 4.972 | 0.127 |
| 4 | 0.272 | 0.233 | 0.253 | 0.107 |
| 5 | 0.233 | 0.183 | 0.208 | 0.170 |
| 6 | 0.196 | 0.205 | 0.200 | 0.032 |
| 7 | 0.160 | 0.175 | 0.167 | 0.063 |
| 8 | 0.215 | 0.257 | 0.236 | 0.127 |
| 9 | 0.292 | 0.208 | 0.250 | 0.236 |
| 10 | 0.310 | 0.286 | 0.298 | 0.058 |
| 12 | 0.169 | 0.123 | 0.146 | 0.223 |
| 13 | 0.211 | 0.179 | 0.195 | 0.116 |
| 14 | 0.078 | 0.093 | 0.085 | 0.120 |
| 16 | 5.218 | 5.403 | 5.310 | 0.025 |
| 18 | 0.206 | 0.178 | 0.192 | 0.101 |
| 19 | 0.133 | 0.132 | 0.133 | 0.004 |
| 20 | 0.140 | 0.147 | 0.144 | 0.036 |
| 21 | 0.114 | 0.094 | 0.104 | 0.136 |
| 22 | 0.144 | 0.112 | 0.128 | 0.176 |
| 23 | 0.093 | 0.076 | 0.085 | 0.141 |
| 25 | 0.099 | 0.082 | 0.091 | 0.132 |
| 26 | 0.076 | 0.065 | 0.071 | 0.109 |
| 27 | 0.079 | 0.076 | 0.078 | 0.024 |
| 28 | 0.133 | 0.134 | 0.134 | 0.006 |
| 29 | 0.151 | 0.148 | 0.150 | 0.014 |
| 30 | 0.100 | 0.091 | 0.095 | 0.060 |
| 31 | 0.103 | 0.110 | 0.107 | 0.042 |
| 32 | 0.063 | 0.088 | 0.076 | 0.242 |
| 33 | 0.059 | 0.085 | 0.072 | 0.250 |
| 34 | 0.085 | 0.076 | 0.080 | 0.081 |
| 35 | 0.093 | 0.102 | 0.098 | 0.062 |
| 36 | 0.096 | 0.106 | 0.101 | 0.067 |
| 37 | 0.113 | 0.140 | 0.126 | 0.153 |
| 38 | 0.136 | 0.162 | 0.149 | 0.126 |
| 39 | 0.061 | 0.075 | 0.068 | 0.145 |
| 40 | 0.135 | 0.124 | 0.130 | 0.061 |
| 41 | 0.104 | 0.126 | 0.115 | 0.134 |
| 43 | 0.131 | 0.141 | 0.136 | 0.052 |
| 44 | 0.451 | 0.471 | 0.461 | 0.030 |
| 45 | 0.049 | 0.064 | 0.056 | 0.176 |
| 47 | 0.068 | 0.059 | 0.063 | 0.107 |
| 49 | 0.088 | 0.115 | 0.101 | 0.188 |
| 50 | 0.094 | 0.079 | 0.087 | 0.124 |
| 51 | 0.227 | 0.216 | 0.222 | 0.034 |
| 52 | 0.165 | 0.138 | 0.151 | 0.126 |
| 54 | 0.179 | 0.164 | 0.172 | 0.065 |
| 55 | 0.131 | 0.102 | 0.116 | 0.175 |
| 56 | 0.194 | 0.147 | 0.171 | 0.197 |
| 58 | 0.172 | 0.173 | 0.173 | 0.002 |
| 59 | 0.289 | 0.296 | 0.292 | 0.017 |
| 60 | 0.139 | 0.151 | 0.145 | 0.060 |
| 61 | 0.217 | 0.235 | 0.226 | 0.057 |
| 62 | 0.177 | 0.172 | 0.175 | 0.021 |
| 63 | 0.148 | 0.158 | 0.153 | 0.045 |
| 64 | 0.182 | 0.136 | 0.159 | 0.202 |
| 65 | 0.245 | 0.191 | 0.218 | 0.175 |
| 66 | 0.153 | 0.155 | 0.154 | 0.010 |
| 67 | 0.183 | 0.177 | 0.180 | 0.025 |
| 68 | 0.103 | 0.191 | 0.147 | 0.426 |
| 69 | 0.253 | 0.359 | 0.306 | 0.245 |
| 70 | 0.151 | 0.153 | 0.152 | 0.008 |
| 71 | 0.767 | 0.568 | 0.668 | 0.211 |
| 73 | 0.191 | 0.155 | 0.173 | 0.145 |
| 74 | 0.135 | 0.131 | 0.133 | 0.022 |
| 75 | 0.324 | 0.347 | 0.335 | 0.049 |
| 76 | 0.179 | 0.163 | 0.171 | 0.064 |
| 77 | 0.348 | 0.260 | 0.304 | 0.203 |
| 79 | 0.136 | 0.105 | 0.120 | 0.183 |
| 80 | 0.313 | 0.278 | 0.295 | 0.083 |
| 81 | 0.179 | 0.162 | 0.171 | 0.069 |
| 82 | 0.098 | 0.098 | 0.098 | 0.003 |
| 83 | 0.103 | 0.117 | 0.110 | 0.088 |
| 84 | 0.165 | 0.148 | 0.156 | 0.077 |
| 86 | 0.322 | 0.307 | 0.315 | 0.032 |
| 87 | 0.337 | 0.304 | 0.320 | 0.074 |
| 88 | 0.277 | 0.263 | 0.270 | 0.035 |
| 91 | 0.222 | 0.202 | 0.212 | 0.065 |
| 92 | 0.217 | 0.198 | 0.208 | 0.064 |
| 93 | 0.130 | 0.123 | 0.127 | 0.039 |
| 94 | 0.256 | 0.297 | 0.277 | 0.104 |
| 95 | 0.271 | 0.249 | 0.260 | 0.061 |
| 96 | 0.244 | 0.210 | 0.227 | 0.108 |
| 98 | 0.161 | 0.137 | 0.149 | 0.116 |
| 99 | 0.376 | 0.333 | 0.354 | 0.087 |
| 100 | 0.215 | 0.224 | 0.220 | 0.029 |
| 102 | 0.261 | 0.244 | 0.252 | 0.048 |
| 103 | 0.179 | 0.169 | 0.174 | 0.040 |
| 104 | 0.267 | 0.276 | 0.272 | 0.023 |
| 105 | 0.253 | 0.250 | 0.252 | 0.007 |
| 106 | 0.249 | 0.234 | 0.242 | 0.044 |
| 107 | 0.298 | 0.295 | 0.297 | 0.007 |
| 108 | 0.328 | 0.336 | 0.332 | 0.017 |
| 110 | 0.110 | 0.094 | 0.102 | 0.110 |
| 111 | 0.197 | 0.182 | 0.190 | 0.054 |
| 112 | 0.341 | 0.330 | 0.335 | 0.022 |
| 114 | 0.189 | 0.179 | 0.184 | 0.038 |
| 115 | 0.152 | 0.178 | 0.165 | 0.111 |
| 117 | 0.199 | 0.151 | 0.175 | 0.194 |
| 120 | 0.469 | 0.334 | 0.401 | 0.237 |
| 121 | 0.206 | 0.370 | 0.288 | 0.402 |
| 122 | 0.128 | 0.139 | 0.134 | 0.057 |
| 123 | 0.020 | 0.001 | 0.011 | 1.312 |
| 124 | 0.348 | 0.308 | 0.328 | 0.086 |
| 125 | 0.274 | 0.265 | 0.270 | 0.024 |
| 126 | 0.119 | 0.166 | 0.142 | 0.237 |
| 127 | 0.065 | 0.115 | 0.090 | 0.394 |
| 128 | 0.148 | 0.169 | 0.158 | 0.093 |
| 129 | 0.315 | 0.490 | 0.402 | 0.308 |
| 130 | 0.211 | 0.294 | 0.252 | 0.232 |
| 131 | 0.382 | 0.379 | 0.380 | 0.007 |
| 132 | 0.138 | 0.156 | 0.147 | 0.083 |
| 133 | 0.531 | 0.363 | 0.447 | 0.266 |
| 134 | 0.168 | 0.155 | 0.161 | 0.059 |
| 135 | 0.326 | 0.326 | 0.326 | 0.000 |
| 136 | 0.501 | 0.417 | 0.459 | 0.129 |
| 137 | 0.142 | 0.194 | 0.168 | 0.219 |
| 138 | 0.427 | 0.315 | 0.371 | 0.215 |
| 139 | 0.226 | 0.208 | 0.217 | 0.057 |
| 140 | 0.481 | 0.429 | 0.455 | 0.081 |
| average CV |  |  |  | 0.115 |

**Supplemental table S7a**: Concordance between QDB and IHC for specimens of unanimous agreement (n=60). The concordance rate was calculated at (42+4)/(42+4+3+1)=92.0%.

|  | IHC 0 | IHC 1+ |
| --- | --- | --- |
| 0_q_ | 42 | 1 |
| E_q_ | 9 | 1 |
| 1+_q_ | 3 | 4 |

**Supplemental table S7b** : Concordance between QDB and IHC for specimens of ≥90 agreement (n=78). The concordance rate was calculated at (54+7)/(54+7+3+2)=92.4%.

|  | IHC 0 | IHC 1+ |
| --- | --- | --- |
| 0_q_ | 54 | 2 |
| E_q_ | 10 | 2 |
| 1+_q_ | 3 | 7 |

**Supplemental table S7c**: Concordance between QDB and IHC for specimens of consensus agreement (n=88). The concordance rate was calculated at (57+10)/(57+10+3+4)=90.5%.

|  | IHC 0 | IHC 1+ |
| --- | --- | --- |
| 0_q_ | 57 | 4 |
| E_q_ | 10 | 4 |
| 1+_q_ | 3 | 10 |

**Supplemental table S7d**: Concordance between QDB and IHC for specimens of ≥50 agreement (n=119). The concordance rate was calculated at (66+13)/(66+13+5+12)=82.3%.

|  | IHC 0 | IHC 1+ |
| --- | --- | --- |
| 0_q_ | 66 | 12 |
| E_q_ | 13 | 10 |
| 1+_q_ | 5 | 13 |

**Supplemental table S8a**: Concordance between QDB and IHC for specimens of unanimous agreement (n=107). The concordance rate was calculated at (69+7)/(69+7+6+1)=91.2%.

|  | IHC 0 | IHC 1+ |
| --- | --- | --- |
| 0_q_ | 69 | 1 |
| E_q_ | 22 | 2 |
| 1+_q_ | 6 | 7 |

**Supplemental table S8b:** Concordance between QDB and IHC for specimens of ≥90% agreement (n=135). The concordance rate was calculated at (89+10)/(89+10+8+2)=90.8%.

|  | IHC 0 | IHC 1+ |
| --- | --- | --- |
| 0_q_ | 89 | 2 |
| E_q_ | 23 | 3 |
| 1+_q_ | 8 | 10 |

**Supplemental table S8c**: Concordance between QDB and IHC for specimens of consensus agreement (≥75%) (n=167). The concordance rate was calculated at (99+16)/(99+16+9+5)=89.1%.

|  | IHC 0 | IHC 1+ |
| --- | --- | --- |
| 0_q_ | 99 | 5 |
| E_q_ | 29 | 9 |
| 1+_q_ | 9 | 16 |

**Supplemental table S8d**: Concordance between QDB and IHC for specimens of ≥50% agreement (n=225). The concordance rate was calculated at (113+26)/(113+26+14+14)=83.2%.

|  | IHC 0 | IHC 1+ |
| --- | --- | --- |
| 0_q_ | 113 | 14 |
| E_q_ | 37 | 21 |
| 1+_q_ | 14 | 26 |
